# STAGdb: a 30K SNP genotyping array and Science Gateway for *Acropora* corals and their dinoflagellate symbionts

**DOI:** 10.1038/s41598-020-69101-z

**Published:** 2020-07-27

**Authors:** S. A. Kitchen, G. Von Kuster, K. L. Vasquez Kuntz, H. G. Reich, W. Miller, S. Griffin, Nicole D. Fogarty, I. B. Baums

**Affiliations:** 1 Department of Biology, The Pennsylvania State University, 208 Mueller Laboratory, University Park, PA 16802 USA; 2 The Huck Institutes of the Life Sciences, The Pennsylvania State University, University Park, PA 16802 USA; 3 Centre for Comparative Genomics and Bioinformatics, The Pennsylvania State University, University Park, PA 16802 USA; 4 NOAA Restoration Center, 260 Guard Rd., Aguadilla, PR 00603 USA; 5 Department of Biology and Marine Biology, Center for Marine Science, University of North Carolina Wilmington, Wilmington, NC 28403 USA

**Keywords:** Bioinformatics, Genetic databases, Data processing, Marine biology, Conservation biology, Ecological genetics, Restoration ecology

## Abstract

Standardized identification of genotypes is necessary in animals that reproduce asexually and form large clonal populations such as coral. We developed a high-resolution hybridization-based genotype array coupled with an analysis workflow and database for the most speciose genus of coral, *Acropora*, and their symbionts. We designed the array to co-analyze host and symbionts based on bi-allelic single nucleotide polymorphisms (SNP) markers identified from genomic data of the two Caribbean *Acropora* species as well as their dominant dinoflagellate symbiont, *Symbiodinium ‘fitti’.* SNPs were selected to resolve multi-locus genotypes of host (called genets) and symbionts (called strains), distinguish host populations and determine ancestry of coral hybrids between Caribbean acroporids. Pacific acroporids can also be genotyped using a subset of the SNP loci and additional markers enable the detection of symbionts belonging to the genera *Breviolum, Cladocopium*, and *Durusdinium*. Analytic tools to produce multi-locus genotypes of hosts based on these SNP markers were combined in a workflow called the Standard Tools for Acroporid Genotyping (STAG). The STAG workflow and database are contained within a customized Galaxy environment (https://coralsnp.science.psu.edu/galaxy/), which allows for consistent identification of host genet and symbiont strains and serves as a template for the development of arrays for additional coral genera. STAG data can be used to track temporal and spatial changes of sampled genets necessary for restoration planning and can be applied to downstream genomic analyses. Using STAG, we uncover bi-directional hybridization between and population structure within Caribbean acroporids and detect a cryptic Acroporid species in the Pacific.

## Introduction

Genotype identification and tracking are required for well-replicated basic research experiments and in applied research such as designing restoration projects. High-resolution genetic tools are necessary for large clonal populations where genets can only be delineated via genotyping. The advent of reduced representation sequencing methods such as Genotype-By-Sequencing (GBS) or Restriction-site Associated DNA Sequencing (RADseq) have made it possible to assay a large number of single-nucleotide polymorphism (SNP) loci in any organism at a reasonable cost^1^. These methods are widely used in population genomics but have the disadvantage that the SNP loci are anonymous. Thus, there is no guarantee that the same set of SNP loci will be recovered from each sample within an experiment or between experiments, making it more difficult to design standardized workflows. To circumvent this issue, standardized SNP probes can be designed for reproducible genotyping and analysis from hundreds of samples using modified RAD-based approaches like Rapture^2^, RADcap^3^, and quaddRAD^4^ or using hybridization-based SNP genotyping arrays. Hybridization-based SNP arrays tend to have lower error rates then RADseq methods^5,6^ and thus increased accuracy of genet identification and tracking. However, both approaches forgo discovery of new SNP loci in favor of assaying a standard set of probes across all samples resulting in some ascertainment bias^7–9^.

When it comes to the analysis of SNP genotyping data, familiarity with computer programming and access to high performance computing is typically required but not always available. Because genotyping arrays contain a known set of SNP loci, standardized workflows can be designed easily. Galaxy is an open source, web-based platform for data-intensive biomedical research^10^ and provides the underlying framework for Science Gateways. Science Gateways are extensions of cyberinfrastructure, like Galaxy, that focus on a specific scientific communities’ needs by providing digital interfaces of computational resources which lowers the barriers (know-how and cost) often associated with these resources. The use of a standardized workflow within a Scientific Gateway enables scientists and restoration practitioners to accurately match samples to existing genets and strains, discover novel genets/strains and track their fate across years, all from a web browser.

Corals, like other clonal plant and animal species, reproduce frequently via asexual fragmentation^11–15^. Over time coral genets can extend over tens of meters consisting of tens to hundreds of colonies^16–18^. This leads to considerable variability in genotypic evenness and richness on small spatial scales, ranging from minimal clonal replication to reefs dominated by a single genet ^12,14,18,19^. The importance of coral genets in explaining variation in growth rates and stress response is becoming increasingly clear^20–24^. Further, hermaphroditic corals species like the Caribbean acroporids are mainly self-incompatible, thereby requiring the presence of gametes from different genets for successful sexual reproduction^25,26^. For these reasons, identification of genets and preservation of genotypic diversity are conservation priorities^27^.

Tropical corals frequently house single-celled photosynthetic algae in the family Symbiodiniaceae that provide the majority of the hosts organic carbon^28,29^. Coral species differ in their symbiont specificity, and colonies may house several algal genera within their cells at a given time. Thus, the complex mixtures of coral and algal DNA present challenges and opportunities for the development of high-resolution co-genotyping methods. Microsatellite markers specific for certain species of algae have further revealed subspecies level strain diversity and elucidated the temporal and spatial dynamics of symbiont strain/host genet associations^30–38^, but no SNP-based markers are available yet. Given that the algal species associated with a coral colony can influence the colony’s physiology, it is also of interest to researchers and practitioners to identify the dominant and any background symbionts in coral samples.

**Figure 1:**
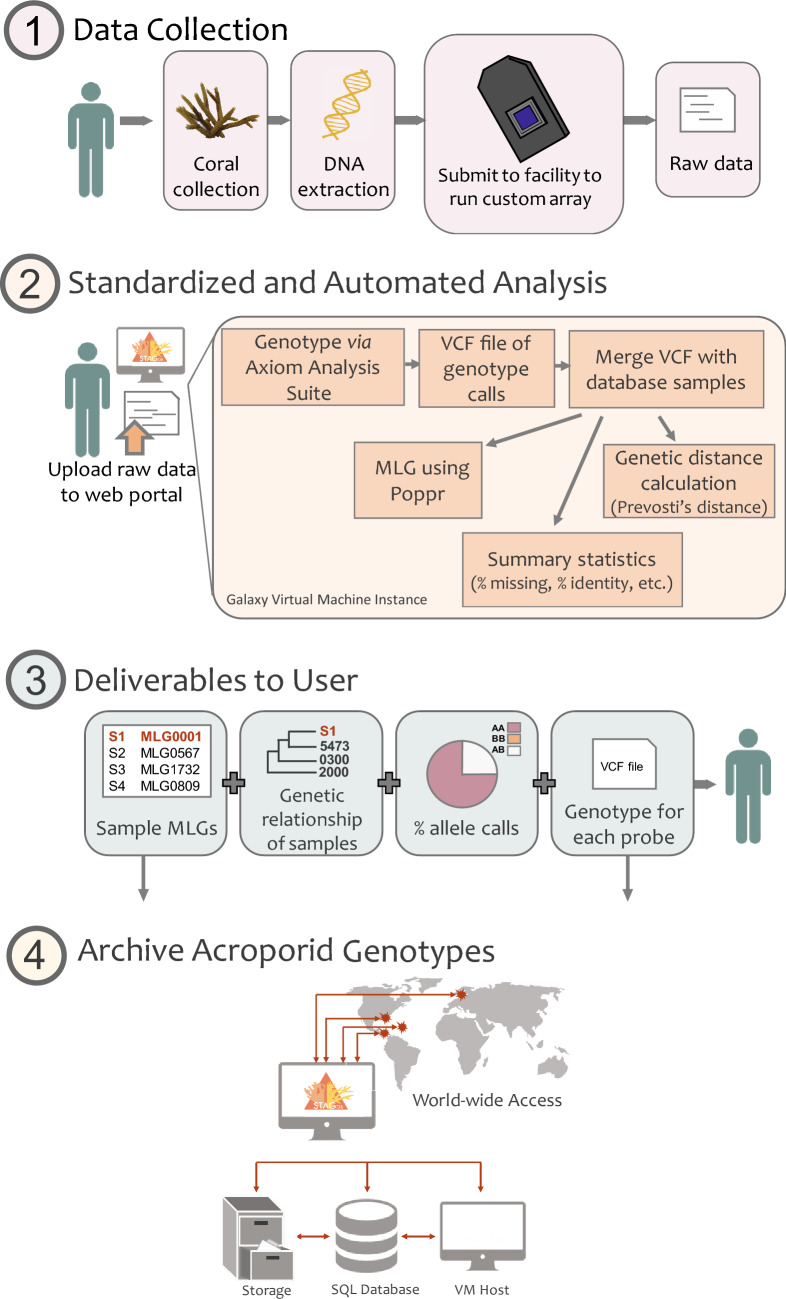
General overview of Standard Tools for Acroporid Genotyping. Step (**1**) user collects the coral, extracts the DNA and submits the DNA to their closest processing facility. Step (**2**) user uploads metadata and raw data to the Galaxy CoralSNP environment for analysis. Step (**3**) user downloads their multi-locus genotypes (MLG) among other deliverables. Step (**4**) the new sample MLGs and genotype information is deposited in the postgreSQL database that can be accessed from anywhere.

Corals often occur in remote locations without access to molecular laboratory and computation facilities or require special export permits to transport tissue samples to well-equipped facilities. Thus, we aimed to develop a genotyping array designed for instruments available at most major hospitals around the world. Genotyping arrays can be processed by a sequencing facility with user supplied tissue (as well as extracted DNA; Fig. 1) eliminating the need for a molecular laboratory and therefore, can be widely adopted by users without access to such facilities.

Here, we report the development of a SNP array and standardized analysis workflow for the most speciose genus of coral, *Acropora*. The roughly 120 *Acropora* spp. dominate shallow reefs in the Pacific and Atlantic oceans^39,40^. In the Caribbean, the primary shallow reef builders are *Acropora palmata* and *A. cervicornis,* which form a hybrid (commonly known as *A. prolifera*)^41–43^. Because of drastic population declines, they are listed as threatened under the U.S. Endangered species act, making them the focal species in reef restoration efforts across the Caribbean. Promoting genotypic diversity within nurseries and outplanting sites is a management priority for these species. We present a ~ 30k SNP genotyping array that identifies host and symbiont genotypes, coral hybrid status and background symbiont genera. The array can be analyzed cost-effectively in a standardized manner using the Standard Tools for Acroporid Genotyping (STAG) within a Galaxy environment (Fig. 1). We further establish a publicly available database of *Acropora* genets. This approach can serve as a template for other asexually producing species of conservation concern.

## Results

### Array design and validation

**Figure 2:**
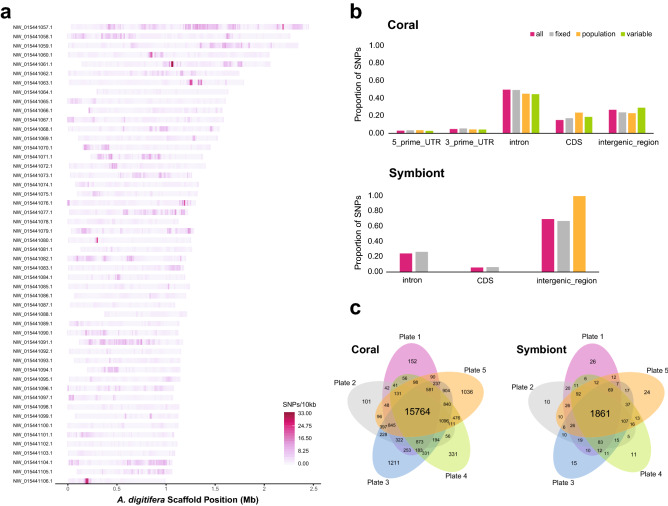
Density, distribution and recovery of SNP probes. The probe density over 10,000 bp windows is mapped onto the 50 longest *A. digitifera* reference scaffolds (**a**). The highest density exceeds 33 probes in a given interval, where most intervals are between 0 to 8 probes. The proportion of designed probes are compared for coding and non-coding regions in the genomes of the coral and symbionts (**b**). All probes are pink, fixed probes are grey, population probes are orange, and variable probes are green. The recommended probes shared between each plate are shown in the Venn diagrams (**c**).

We identified 1.6 million high-quality coral SNPs that varied between the genomes of 42 previously sequenced *A. palmata* and *A. cervicornis* from four locations (Belize, Curacao, Florida, and U.S. Virgin Islands) using two variant callers, samtools mpileup^44^ that uses likelihood scores and freebayes^45^ that uses Bayesian posterior probabilities for variant calls. After Affymetrix filtered the 34,783 coral loci, the final array contained 32,124 loci with 53,579 probes, broken down into 25,889 fixed, 17,803 population and 9,887 *A. palmata* variable probes (Table S1 and Fig. 2). The majority of these variable sites are found within introns of coding sequences in the *A. digitifera* genome, followed by intergenic regions (Fig. 2b).

When comparing two deeply-sequenced *A. palmata* and *A. cervicornis* genomes to the reference *S. tridanidornium* genome, we identified 2,657 high-quality symbiont SNPs using samtools mpileup^44^. When comparing 42 coral genome samples including the two above^46^ to the draft genome of *A. cervicornis* ‘like’ *S. ‘fitti’*, 60,946 SNPs were considered high-quality (Reich et al., In Prep). Applying similar filtering methods to identify so-called ‘fixed’ differences between strains and populations as was done in the coral, we were left with only a small fraction of SNPs. Given the status of the *S. ‘fitti’* genome analysis at the time of the array design, we submitted more probes from the first comparison than the latter (2,269 from first comparison and 380 from the second comparison). Those loci were mostly found in the intergenic regions of the *Symbiodinium* genomes (Fig. 2b). Of the 2,661 symbiont loci we submitted, all were retained in the final array with 4,021 probes covering fixed (*n* = 3,663), population (*n* = 304) and genera (*n* = 54) categories (Table S1).

The recommended coral probes from the first plate were designated as the genotyping probes for the Caribbean acroporids in all subsequent analyses (Fig. 2c and Table S2). For the symbionts, all samples from the five plates that passed quality filtering (*n* = 293 samples) were re-analyzed together using the ‘Best Practices Workflow’ (BPW). The recommended probes were reduced further after removing probes that matched draft genome assemblies of *A. palmata* (Kitchen, unpublished), *A. cervicornis* (Kitchen, unpublished), *A. tenuis* ^47^, *A. hyacinthus* ^47^, and *A. millepora* ^48^ with high homology (blastn, e-value 1e−13), were not classified as Poly High Resolution, and had limited resolution outside of Florida samples (see Table S3). In particular, there were 146 probes that only distinguish the deeply-sequenced *A. cervicornis* symbiont strain, 247 probes that only distinguish the deeply-sequenced *A. palmata* symbiont strain, and 944 probes that distinguish the Florida *A. cervicornis* symbiont strains (*n* = 36 samples) from all the other samples. This resulted in 531 symbiont genotyping probes for downstream analysis.

The genotype success for each plate is presented in Table S4. The quality was first assessed by the background fluorescence of the non-polymorphic probes, or dish quality with a threshold of 82%. Then, only the samples with a call rate of 97% for the coral or symbiont probes, respectively, proceeded to the next step in the analysis. Because some of the samples were symbiont-enriched DNA or exclusively symbiont culture DNA, they failed BPW for the coral probe set. Alternatively, coral sperm and larvae failed the symbiont probe set (Table S4). Overall, Caribbean coral genotype calling was successful for samples with DNA concentrations as low as 0.064 ng/µl and as high 203.34 ng/µl (Table S5). Symbiont genotype calling worked for samples with DNA concentrations ranging from 0.23 to 203.34 ng/µl (Table S5).

### Coral genotyping via analysis portal

**Figure 3:**
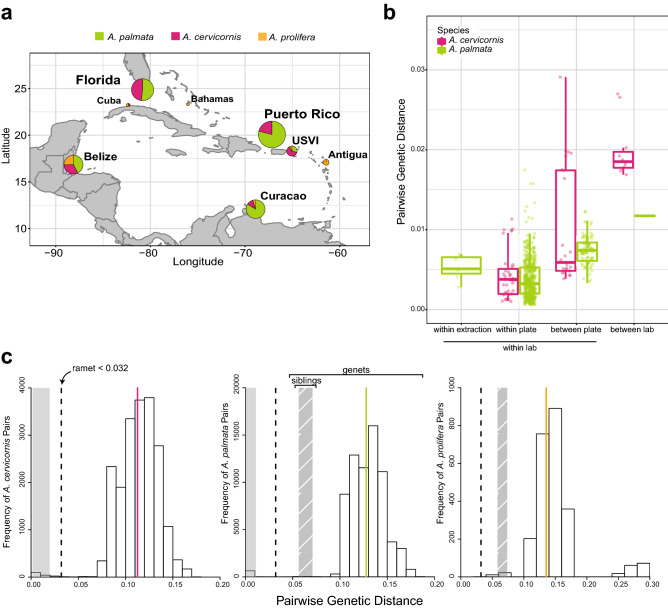
Caribbean acroporid genet identification. Pie-charts on the map of the Caribbean represent the percentage of species at each collection location for the 479 genotyped samples (**a**). Prevosti’s pairwise genetic distance of ramets, or clone mates, was compared between technical replicates, samples within a plate and samples between plates processed within the same laboratory to those processed in a different laboratory (**b**). A histogram of the frequency of pairwise genetic distance values for each species indicates a break between ramets and genets (**c**). The dashed line is the threshold for ramet identification and the solid line is the average genetic distance for genets in the taxon (pink = *A. cervicornis*, green = *A. palmata*, orange = *A. prolifera*). The solid grey and hatch-marked grey shaded areas represent the mean ± standard deviation for ramets and siblings for each taxon, respectively.

Four hundred seventy-nine corals (out of 520) were successfully genotyped using the genotyping probe set (Table S4 and Fig. 3a) in the Galaxy CoralSNP analysis environment. The missing data ranged from 0.06% to 3.22% for the samples analyzed on the array (Fig. S1). Plates differed in the amount of missing data that we attributed to a batch effect of sample preparation, but not sample preservative or extraction method because these were shared between plates. A significant positive correlation was detected between percent missing data and percent heterozygosity for each species (Pearson’s Correlation, *A. palmata* R^2^ = 0.4507, *p* = 8.142e−14; *A. cervicornis* R^2^ = 0.8223, *p* < 2.2e−16; Fig. S2a), both of which are indications of sample quality. Misclassification of heterozygous calls can occur in samples with lower quality^49,50^.

Technical variation between replicate runs of the same DNA was low with an average genetic distance of 0.0053 ± 0.0015 between technical replicates (Mean ± 1 SD; samples SI-1, SI-10, SI-12, Table S6 and Fig. 3b). The average pairwise genetic distance of ramets from the same genet (clone mates) within a plate was 0.0038 ± 0.0026 and between plates was 0.0079 ± 0.0041 (Fig. 3b). Due to the larger genetic distances between technical replicates than ramets observed within a plate, we tested for differences in the five plates. There was a significant effect of plate on the genetic distance of ramets analyzed within plate (1-way ANOVA, F (4,391) = 17.58, *p* = 2.81e−13). Plate 9SR22843, which contained the technical replicates, had larger average pairwise genetic distances between ramets and technical replicates within the plate compared to three of the other plates (Tukey HSD, 9SR22843 was on average 0.0014 larger than 9SR22844 *p* = 0.0003; 9SR22843 was on average 0.0015 larger than P9SR10073 p = 0.0019; 9SR22843 was on average 0.0025 larger than P9SR10076 *p* = 0.0000).

The threshold for genet assignment of samples was defined using previously identified ramets, ranging from two to six ramets per genet (shared baums_coral_genet_id in Table S5). The largest genetic distance within known ramets was ca. 0.0312 between a genome sample and array sample (ie. 14120_Mixed and 4960, Table S7). We used pairwise genetic distance = 0.032 as the threshold for genet assignment based on the observations above (Fig. 3c). The average pairwise genetic distance among ramets was 0.0064 ± 0.0064 for all genet IDs with more than one ramet and ranged from 0.0006 to 0.0312 (Table S7). Additionally, tissue from eight genets extracted in two different laboratories recovered the same genet ID, albeit with differences in DNA concentration, missing data, and percent heterozygosity of the fixed probes (Fig. 3b and Table S8). There was between 0.012 to 0.027 pairwise genetic distance among ramets of the same genet in this set, which is like what was observed for differences in genotyping methods (genome sequencing vs. array) and is within the genet threshold.

Between genet pairwise distance was on average 0.113 ± 0.023 for *A. cervicornis* and 0.128 ± 0.025 for *A. palmata* (Fig. 3c). In the case of siblings from outcrossed offspring, the genetic distance ranged from 0.047 (SWSA-140 and SWSA-124) to 0.078 (SWSA-105 and SWSA-128) with an average genetic distance of 0.0642 ± 0.0068 (Fig. 3c). Heterozygosity also varied by species and geographic region, ranging from 0.110 to 0.127 in *A. cervicornis* and 0.132 to 0.156 in *A. palmata* (Table 1 and Fig. S2b). The inbreeding coefficient F_IS,_ which calculates the proportion of alleles within an individual that are shared with the population, was highest in Belize and Florida in both species (Table 1).

**Table 1:** Summary of population genetic variation of Caribbean acroporids estimated with 19,694 genotyping probes.

Species	Population	N	N_G_	H_O_	H_S_	F_IS_
*A. cervicornis*	Belize	27	18	0.117	0.122	0.033
	Cuba	1	1	NA	NA	NA
	Curacao	9	7	0.126	0.128	− 0.002
	Florida	54	46	0.110	0.113	0.038
	Puerto Rico	35	21	0.127	0.118	− 0.042
	USVI	16	9	0.113	0.109	− 0.023
*A. palmata*	Belize	37	27	0.148	0.149	0.013
	Curacao	73	57	0.132	0.124	0.013
	Florida	58	26	0.151	0.154	0.021
	Puerto Rico	132	75	0.141	0.140	0.000
	USVI	8	8	0.156	0.156	− 0.004
*A. prolifera*	Antigua	8	8	0.656	0.410	− 0.543
	Bahamas	2	2	0.692	0.415	− 0.705
	Belize	21	21	0.674	0.406	− 0.580
	Cuba	2	2	0.679	0.415	− 0.673
	Curacao	4	4	0.689	0.382	− 0.770
	USVI	2	2	0.700	0.412	− 0.725

*N* number of samples, *N*_*G*_ number of genets, *H*_*O*_ average observed heterozygosity, *H*_*S*_ average expected proportion of heterozygote individuals in the subpopulations, *F*_*IS*_ average inbreeding coefficient.

**Figure 4:**
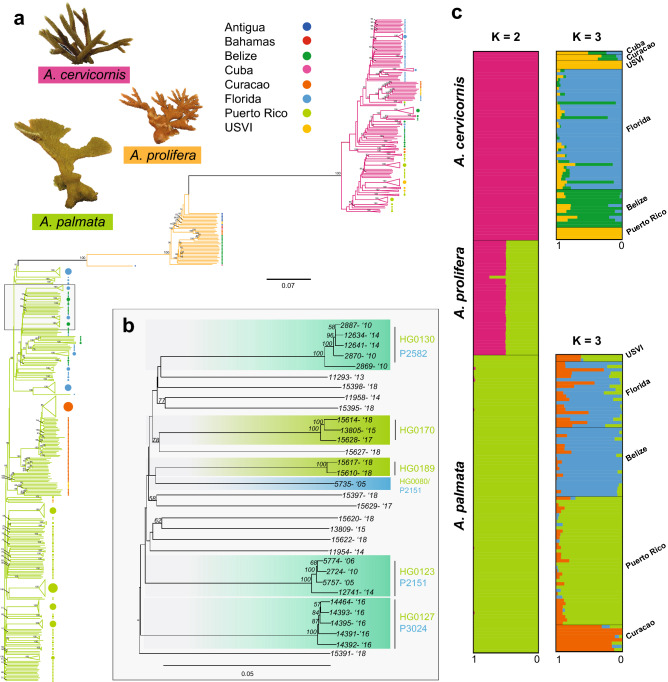
Caribbean acroporid population analysis. Prevosti’s genetic distance of 19,694 SNPs was used to construct a neighbor-joining tree (**a**). The branches are colored by their genetic species identification and collection locations are indicated by the color of the circle at the terminal ends (Antigua = blue, Bahamas = red, Belize = green, Cuba = pink, Curacao = orange, Florida = light blue, Puerto Rico = light green, and USVI = yellow). Nodal support is based on the 100 bootstrap replicates. The nodes of genets with multiple ramets identified with the SNP data are collapsed in the tree. An example of genet resolution is provided based on the array SNP data and the previous microsatellite IDs over different collection years (**b**). The SNP genet ID is presented in green on the top and the microsatellite genet ID is presented in blue on the bottom. The clades are shaded blue-green where the two genotyping methods are congruent. The collection year is presented next to the sample identification number (- ‘xx). ADMIXTURE was run on a representative sample for each genet (*n* = 193), excluding genome samples, offspring of a Curacao cross and Puerto Rico samples from plate 9SR22844 (**c**). Individual bars represent the relative proportion of membership of a sample to the inferred K populations. Results from two source populations for all samples and three source populations for each species separately (K = 3 had the lowest cross-validation error for both species, Fig. S4).

Genet resolution was reproducible across collection years, plates and different laboratories (Figs. 3b, 4 and Table S8). For example, HG0127 and HG0170 were recovered from samples collected between 2005 to 2018 and run on two different plates (Fig. 4b). There was only one case where a genet defined via microsatellite genotyping was split into two genets as defined via SNP genotyping (blue lineage in Fig. 4b). In the inverse situation, there were four cases where genets defined via microsatellite genotyping were no longer considered to be unique genets and combined with other samples defined via SNP genotyping (Table S5).

A Neighbor-Joining tree (Fig. 4a) using the Prevosti’s genetic distance and identity-by-state analysis (Fig. S3) clustered the samples, first by species and then by their collection location. However, the geographic regions were not clearly delineated using these methods. We could recover population clusters using an unsupervised model-based approach with ADMIXTURE (Fig. 4c). After genet correction and applying a minor allele threshold of 5%, 18,823, 7,019, and 6,097 coral loci remain for all three taxa (n = 193 samples), *A. palmata* (n = 90 samples) and *A. cervicornis* (n = 64 samples), respectively. The ancestry of each sample was assessed assuming two source populations for the full dataset and two to ten populations for each species separately. For K = 2 of the entire dataset, the two species clearly separate with the hybrids having mixed ancestry (Fig. 4c). The lowest prediction error for *A. cervicornis* was three inferred populations (Fig. S4) with a population in Florida, a population in Belize and a population in USVI and Puerto Rico (Fig. 4c). Three populations were also predicted in *A. palmata* with a population in Florida and Belize, a population in Puerto Rico and a population in the Curacao (Fig. 4c).

### Hybrid identification

The genetic species assignment was based on 9,072 fixed probes. The proportion of ancestry from each parental species was calculated for each sample and used to identify hybrids (Fig. S5). There were 39 *A. prolifera* hybrids of which all but one appears to be a F1 hybrid (Fig. 4c and Fig. S5). Based on the field calls, one hybrid detected with the array data was previously misidentified as *A. palmata* and 11 samples identified as hybrids in the field (*n* = 7 larvae and *n* = 4 adults) were assigned to one of the parental species instead.

### Symbiont genotyping

There were 293 samples that passed the BPW for the symbiont probes. Unlike the coral samples, the extraction method mattered for symbiont DNA recovery and genotyping. This is exemplified by the failure of all but one replicate DNA extractions using the magnetic bead protocol and successful genotyping of all samples after DNA extraction with the QIAGEN DNeasy kit. One hundred and eighty six putative *S. ‘fitti’* strains were identified based on a genetic distance threshold of 0.0018. We call these putative strains based on the limited a priori information available for setting the strain detection threshold. Enriched symbiont DNA and mixed DNA extractions from the same tissue shared the same strain ID as did technical replicates of the same DNA extractions from the same ramet (Table S5).

Sometimes more than one strain can be present in a given host and the strain ID might represent a mixture of different *S. ‘fitti’* strains. We attempted to identify colonization of single or multiple strains in a host sample through various supervised and semi-supervised classification methods using the signal intensity of the symbiont genotyping probes. The posterior probabilities of the linear discriminant analysis (LDA) were used to determine likely colonization status for the known and unknown samples (Fig. S6a). There was a difference in the distribution of the multiple and single colonized samples on LD1 (Fig. S6a); however, two single strain samples overlapped the distribution of samples with multiple strains. More unknown samples overlapped with the distribution of samples with multiple strains compared to the distribution of samples with a single strain (Fig. S6a). The decision tree had an accuracy of 53.6% and only required signal intensity of two probes for the classification with the lowest cross-validation error (probes AX.197983721.B and AX.198082605.A, Fig. S6b). For the random forest model, the accuracy was estimated to be 66.9% with higher classification error for the single strain samples (multiple error = 29.4%, single error = 54.5%). Five trees were predicted to have the lowest error with the largest number of nodes, one of which is presented in Figure S6c. Naïve Bayes had an accuracy of 69.2% for the training data. Lastly, the semi-supervised k-nearest neighbor model had an accuracy of 65.8%. The results of all classification models were calculated as the percent agreement of multiple strains prediction (ex. 2 out of 5 tests predicted multiple strains = 40%). There were 112 samples that were likely colonized by a single strain (0–20% agreement for multiple) and 157 samples that were likely colonized by multiple strains (80–100% agreement for multiple) (Table S5).

**Figure 5:**
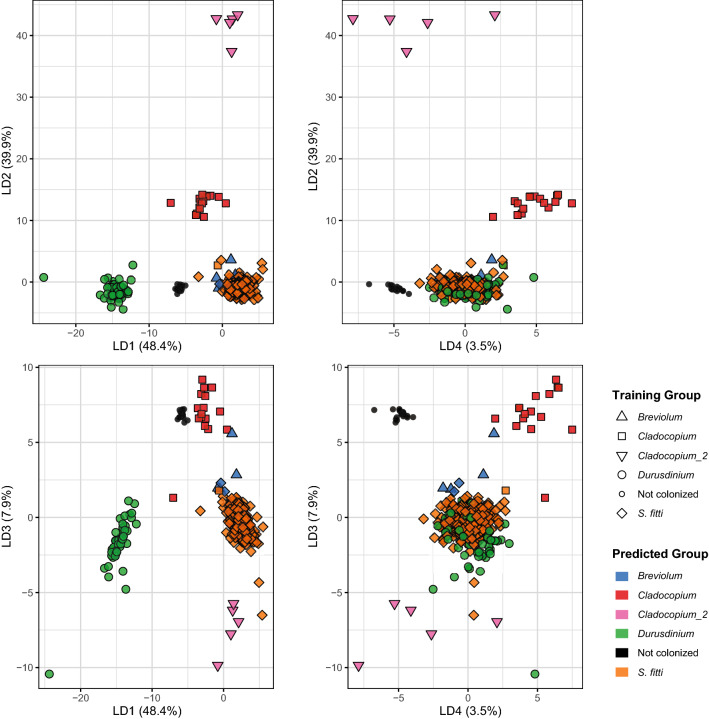
Detection of background symbiont genera. Results of the linear discriminant analysis where the shape denotes the preliminary training group genera assignment and the color is the predicted genera assignment. LD1 separates *Durusdinium* and not colonized samples, LD2 separates *Cladocopium* taxa and LD3 starts to separate *Breviolum* from the *Symbiodinium* group.

In addition to multiple strains of *S. ‘fitti’* present in a single coral host, the coral can be colonized by additional symbiont genera. We used the same classification methods above to detect background genera using the signal intensity of 18 genera probes (Table S9), but each sample was pre-assigned to a genus or classified as not colonized based on their allele patterns. The prediction accuracy of the LDA (Fig. 5), decision tree (Fig. S7a) and random forest (Fig. S7b) was 98.9%, 96.4% and 98.9%, respectively. The predictions for each model are presented in Table S5. The presence of *Breviolum* was detected in thirteen samples with one of the classification methods, ranging from 0.2% to 100% probability. Of these, seven had probabilities greater than 60% and two of those also had *S. ‘fitti’* strain IDs indicating co-infection. The *Cladocopium* containing samples were split into two clusters, one contained samples that were exclusively *A. muricata* hosts (Cladocopium 2) and the other contained host samples that were *A. cervicornis* (*n* = 2), *A. digitifera* (*n* = 8), and *A. millepora* (*n* = 5). Finally, there were 49 samples with *Durisdinium* (*n* = 5 *A. muricata*, 3 *A.cervicornis*, 41 *A. palmata*). Samples containing either *Cladocopium* or *Durisdinium* failed the *S. ‘fitti’* genotyping analysis.

### Suitability for Pacific acroporids

**Figure 6:**
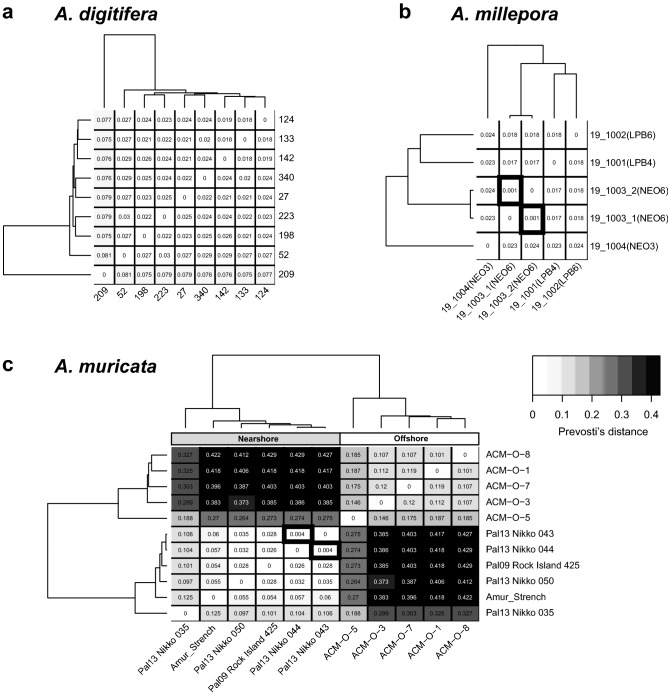
Genetic distance of Pacific acroporids using 1,779 shared probes. The relatedness of samples from three Pacific species, *A. digitifera* (**a**), *A. millepora* (**b**) and *A. muricata* (**c**) were compared using Prevosti’s genetic distance. The distance for each pairwise sample combination is displayed in the respective square of the heatmap. The darker the shading, the larger the genetic distance between samples. The dendrogram on the top and side represents the hierarchical clustering of the samples based on genetic relatedness. Samples with thick black borders are nearly identical for the probes tested and are likely the same genet. In the case of *A. muricata* (**c**), clear separation is observed of nearshore and offshore samples.

Based on in silico genome searches, 26,963 of the coral probes matched *A. hyacinthus*, 28,395 matched *A. millepora* and 14,399 matched *A. tenuis.* Given that our probes were designed using the genome assembly of *A. digitifera* and that they had high homology to other species, we tested whether we could find a conserved set of probes across the Pacific acroproids for future genotyping studies. The Pacific samples were run separately for each species in the genotyping mode in the Axiom Analysis Suite to get the recommended probe sets. This analysis did not enforce a dish-quality threshold. A total of 15,717, 21,520 and 7,275 probes were recommended for *A. digitifera* (*n* = 9 samples), *A. millepora* (*n* = 5 samples) and *A. muricata* (synonom = *A. formosa*; *n* = 11 samples), respectively. Only those probes that were recommended for all three species were used for further analysis (*n* = 1,779 probes, Table S10). The pairwise genetic distance among *A. digitifera* samples ranged from 0.018 to 0.081 (Fig. 6a), with tight clustering in all but one sample. Two *A. millepora* samples were nearly identical (Prevosti’s distance = 0.00084) and differed only at two probes (Fig. 6b), while the largest pairwise genetic distance was only 0.024 (difference of 42 probes). Similarly, two *A. muricata* samples were also closely related, with a Prevosti’s distance of 0.004 (Fig. 6c). For this species a clear pattern emerged separating the nearshore and offshore samples with a maximum pairwise distance of 0.429 (763 probes, Fig. 6c). Although the sample size is too limited for each species to determine genotyping thresholds, less than 50 loci are necessary to identify the 33 unique genets in this dataset based on a genotype accumulation curve (Fig. S8).

## Discussion

Here we report the first genotyping array for corals, which in combination with an open access Galaxy Scientific Gateway to execute the Standard Tools for Acroporid Genotyping (STAG) workflow produces multi-locus genotypes for coral hosts and their algal symbionts. In the workflow, new user-supplied samples are compared to previously genotyped samples and their results contribute to the growing STAG database (Fig. 1). This archive of coral genets and symbiont strains can be used to identify reefs with high host and/or symbiont genetic diversity, temporal and spatial changes, and shuffling in host-symbiont pairings. In addition, a subset of the Caribbean genotyping probes can be used to genotype Pacific acroporids, expanding the utility of the STAG workflow to hundreds of species.

The SNP array and analysis workflow developed here delineate genets in agreement with the previous gold standard for Caribbean acroporid genotyping, multiplex microsatellite genotyping^25^. The STAG workflow uses 61% of the coral loci to produce the host genotype (Table S1) and identified 325 genets out of 479 genotyped samples (Table S4). The average genetic distance of 0.0064 (difference of 0.64%) among ramets was well below our maximum between genet genetic distance threshold of 0.032 (Fig. 3c), which accounts for both biological processes (mutations) and technical error during genotyping. We estimate that technical error accounts for ≤ 0.0053 (16.56%) of this variation based on the lower genetic distance observed within plate for both species than the replicate analysis on the same DNA extraction from a single tissue sample (Fig. 3b and Table S6). The differences observed in ramet genetic distance between plates may be due to the genotyping probe set applied to all plates irrespective of the recommended set for each plate (Table S2). Differences in genetic distances of ramets can also arise from DNA quality that is influenced by sample preservation, tissue type, extraction method, and extraction laboratory. We found a positive relationship between missing data and total heterozygosity (Fig. S2), suggesting that a portion of heterozygous genotype calls in the lower quality samples might be an artifact of technical error. This was evident in the different percent heterozygous estimate of the fixed probes in the between laboratory replicate extractions (Table S8). However, our technical error is similar to previous genotype concordance estimates ranging from 0.2% to 2.4% for replicates of a given subject genotyped on Affymetrix SNP arrays for humans^50^, rainbow trout^6^, soybean^51^ and walnut^52^. In that latter study, the variation was also higher between technical replicates than biological replicates, which the authors attributed to DNA quality. All these sources of technical variation are accounted for in the genotype assignment by the STAG workflow, resulting in robust coral genet identification.

Technical variability can be minimized by standardizing procedures. We recommend that adult samples of at least 3- 4 polyps are preserved in 95% non-denatured ethanol (190 proof), stored as cold as possible and extracted using the Qiagen DNeasy tissue extraction kit. DNA requirements are modest for the Axiom SNP array. Adult tissue, single larva and concentrated sperm were successfully genotyped in samples with DNA concentrations as low as 63 pg/µl, although higher concentrations are recommended. While high-quality, non-degraded DNA provided the best results, moderately degraded samples (i.e. extractions that show a dense band of high molecular weight DNA with some smearing across size ranges) were also successfully genotyped. DNA requirements with respect to quality and quantity are thus comparable to RADseq and whole genome sequencing techniques.

*A. palmata* and *A. cervicornis* differ in the scale of dispersal with *A. cervicornis* showing higher levels of population subdivision across the Caribbean and North Atlantic compared to *A. palmata*^34,53–57^. *A. palmata* stands were found to be structured into two long-separated East/West populations based on microsatellite data^58^, but additional samples from the Mesoamerican Reef Tract^57^ and the development of SNP markers^59^ resulted in the discovery of further population structure. Our results from a limited number of geographic locations identified three populations in *A. palmata* consistent with the previous study by Devlin-Durante and Baums^59^, recovering the East/West divide with additional substructure between Puerto Rico and Curacao in the East. We also recovered three populations in *A. cervicornis*, but with substructure detected between the Western Caribbean populations of Florida and Belize.

Quantifying the extent to which introgression has historically occurred and may occur now can elucidate the evolutionary and ecological significance of hybridization in acroporids. Using the species-specific fixed SNPs, we identified 39 F1 hybrid genets and corrected several species misidentifications in the field based on colony morphology (one classified hybrid identified as *A. palmata* in the field and two classified *A. palmata* identified as hybrid in the field). While F1 hybrids are more common, later generation backcrosses do occur^41,42^ albeit the direction of introgression has been debated^42,60,61^. Here, we identified one later generation hybrid that was classified as a putative backcross *A. palmata* (44.98% heterozygous and 52.7% *A. palmata;* Fig. S5) in contrast to earlier findings that backcrosses are restricted to introgression of *A. palmata* genes into the *A. cervicornis* genome. A recent report also found putative *A. palmata* backcrosses based on microsatellite data in the Lesser Antilles^62^. Together, these results support the conclusion of bidirectional introgression in Caribbean acroporids.

Because of the intimate association between corals and algae, the SNP array was designed to assay host and symbiont DNA simultaneously, a novel application for the Axiom SNP array. The array contains a much smaller number of symbiont-specific probes compared to host probes and thus information gleaned from these probes is more limited. The large genome size, haploidy and asexuality of *Symbiodinium ‘fitti’*, the dominant symbiont of the Caribbean acroporids^33^, presents challenges. The lower allelic diversity of *S. ‘fitti’* microsatellite loci compared to the allele diversity of their cnidarian host counterparts necessitates using larger number of loci for strain resolution^34^. After exhaustive filtering of the symbiont genotyping probes based on their performance, only 20% of the loci remained which recovered reproducible strain identity in replicate ramets of a given genet. However, given the limited prior strain information for the samples, the conservative threshold we used for strain assignment will need to be validated with more known strains in the future. Only 58% of coral samples with symbionts yielded an *S. ‘fitti’* genotype. Failures were either due to inefficient symbiont DNA recovery in the extraction or to presence of other Symbiodiniaceae genera. Comparison of strain resolution achieved with the SNP array relative to microsatellite strain resolution revealed previously unresolved strain diversity. However, it is not yet clear how much of this strain diversity results from mutational processes versus diversity produced as a result of recombination between strains^34,63^.

*Acropora* colonies are at times colonized by more than one strain of *S. ‘fitti’*^34^ but classification of colonies as being colonized by a single or multiple strains was challenging (Fig. S6). In contrast, the ability to detect the presence of other Symbiodiniaceae genera within coral samples is encouraging (Fig. 5). We detected eight *A. cervicornis* and 44 *A. palmata* colonies that harbored symbionts of the genera *Breviolum*, *Cladocopium* or *Durusdinium*. Of these, three *A. cervicornis* and three *A. palmata* are likely to be co-colonized by *Breviolum* and *S. ‘fitti’*, a combination of symbionts shown to be intermittent in *A. cervicornis* through profiling the *ITS2* region^64^. Further, symbiont genera detected in nearshore (= *Durusdinium*) and offshore (= *Cladocopium*) *A. muricata* samples were consistent with a recent study by Hoadley, et al.^65^, although this taxon of *Cladocopium* (Cladocopium_2) was distinctly different from the other *Cladocopium* taxa (Cladocopium) containing both Caribbean and Pacific hosts (Fig. 5). The two *Cladocopium* groups differed in their signal intensities for the genera probes with samples in the Cladocopium_2 having signal intensity on average 4.5 × higher than samples within the Cladocopium group. Signal intensities may vary due to quantity of DNA, random difference in hybridization efficiency, and variable affinity of probes to different symbiont taxa within genera. Thus, we stress here that the SNP array cannot be used to derive quantitative differences among symbiont taxa associated with a coral sample. Moreover, DNA from cultured *S. tridacnidorium* was also on average 4 × higher than mixed *Acropora*-*S. ‘fitti’* samples, suggesting that “pure” symbiont DNA extracts cannot be directly compared to mixed host-symbiont samples. Further experiments should benchmark the method by testing mixtures of Symbiodiniaceae genera with known composition.

Application of the current array to non-target Pacific acroporid species is possible when the sole intent is to delineate genets as is often required in restoration settings. Using a common set of acroporid probes, we genotyped three additional acroporid species and could identify members of the same genet. Interestingly, we observed genetic distances between *A. muricata* nearshore and offshore samples (0.405 ± 0.017) that were only slightly lower than those observed between *A. palmata* and *A. cervicornis* (0.684 ± 0.051), suggesting potential cryptic speciation between the two divergent *A. muricata* populations. Polyphyletic relationships between conspecific *A. muricata* have been observed^66^ as well as lack of fertilization among morphotypes^67^. To establish a robust threshold for genet assignment in Pacific acroporids, it will be necessary to add additional samples to the STAGdb that have also been genotyped using highly polymorphic microsatellite markers. However, because of the large ascertainment bias inherent in applying probes designed for Caribbean acroporids to long-separated Pacific species, population genetic models and models designed to detect loci under selection should not be applied to this data.

The combination of the tools presented here provides reliable, standardized identification of host genotypes in diverse *Acropora* spp. and symbiont strains of the Caribbean species. These markers and analysis tools can be used for basic research questions such as gene by environment interactions, hybridization history, or identification of loci under selection. Genetic linkage maps can be generated and inbreeding levels, and relatedness questions can be addressed. Because of the low error rate, the SNP array is particularly suited for the detection of somatic mutation, which are expected to be common in the large, old genets that are now dominating Caribbean *Acropora* populations. Restoration practitioners can use the information to design propagule transfer zones and choose genets for nursery rearing.

## Materials and methods

### Coral SNP selection

Coral samples were pulled from an archival tissue databank from previous collection efforts. Sample information can be found in Supplemental Table S4 and in the Supplemental Methods. DNA extraction methods varied depending on the tissue type, sample preservative and laboratory described in the Supplemental Methods. Protocols for sample collection (10.17504/protocols.io.bec8jazw) and DNA extractions (10.17504/protocols.io.bgjqjumw) are available. We previously identified 8.4 million SNPs between the two Caribbean acroporids and the Pacific acroporid *A. digitifera,* and of those 1.6 million high-quality SNPs varied between the Caribbean acroporids^46^. To create a conservative set of SNP, we additionally called variants with freebayes v1.1.0-50-g61527c5^45^ using the same alignment file from the previous study and identified shared SNPs between the two variant callers with vcf-compare v0.1.14-12-gcdb80b8^68^. From these shared SNPs, they were further refined into three informative categories: fixed, population and variable. The “fixed” SNPs are those variants where all 21 individuals of a given species share a nucleotide and the other 21 individuals of the other species share a different nucleotide. The fixed SNPs were filtered to a sample read depth of ≥ 3 and a minimum distance of 500 bp. We also retained those that we previously defined as PCR-ready (*n* = 894, no observed SNPs, indels, low-complexity DNA or unassembled regions within 50 bp on either side of the SNP (see Kitchen, et al.^46^). Population SNPs were identified based on pairwise comparisons of the four different collection sites (Table S11). These SNPs were filtered such that all samples from one site shared an allele with a frequency of 0.8 or greater and differed from the samples of the other site with the alternative allele at a frequency of 0.8 or greater. Finally, variable SNPs were identified by filtering the SNPs to a sample read depth of ≥ 4, allowing no ambiguous bases or repetitive sequences in 71 bp of flanking sequence, a minimum distance of at least 1,000 bp between surrounding SNPs, and an allele frequency between 0.5 and 0.7 for all 21 *A. palmata* samples while the variants was also observed in the *A. cervicornis* samples. SNP frequencies were calculated using –freq parameter with VCFtools^68^.

For each SNP, 35 bp of identical flanking sequence between the species was pulled from the *A. digitifera* genome assembly (NCBI: GCF_000222465.1; Shinzato, et al.^69^) using bedtools getfasta^70^*.* These 71 nucleotide (71mer) candidate sequences were filtered through a series of similarity searches to reduce non-specific sequence capture. First, the sequences were compared to the *A. digitifera* genome assembly using BLAST v2.6.0 (task = blastn, e-value = 1e−13) to determine whether redundant genomic targets were present. Sequences were discarded that had a ≥ 30 bp match with more than one genomic location. To check for repetitive probes, a same-strand self-analysis was performed using blastn (filter query sequence = false, word size = 11, -dust no, e-value = 1e−13, strand = both).

In addition to the SNP probes, we identified non-polymorphic sequences from acroporids by extracting high-quality SNPs that were identical between the two Caribbean acroporids and different from *A. digitifera*. We required a sample read depth of ≥ 6 reads with a minimum distance of 1,000 bp between SNPs and no repetitive or ambiguous bases in the 35 bp flanking sequence. We discarded probes that had significant overlap to the array probes (task = blastn, e-value = 1e−13) and randomly selected 3,000 to act as the background probes.

### Symbiont variant calling and SNP selection

SNP discovery in the symbionts was accomplished by comparing our genome samples to two reference genomes, either the assembly of cultured *S. tridacnidorum* (NCBI: GCA_003297005.1^71^) or partial assembly of the predominant symbiont of *A. palmata* and *A. cervicornis*, *S. ‘fitti’* (Reich et al., unpublished), both of which belong to the genus *Symbiodinium* (ITS2-clade A3). Only 15–25% of the reads mapped to the symbiont genomes, reducing our ability to identify comparable number of SNPs in the symbiont as the coral. Some of the *Symbiodinium* SNPs were identified by comparing only the deep-coverage metagenome sequences of *A. palmata* and *A. cervicornis* to the genome assembly of *S. tridacnidorum*^71^. These SNPs were identified as fixed between the two representative Florida acroporids sampled, but it was unclear if they were fixed between the symbiont strains of the two coral species across their geographic range, just in Florida or just between these two samples. The other *Symbiodinium* SNPs were identified by mapping the 42 re-sequenced genome samples to a draft genome assembly of *S. ‘fitti’* and processed as described in Kitchen, et al.^46^. High-quality SNPs were had a quality phred score > 200 and no more than 20% missing data at a given site among all samples. The 71 bp flanking sequences were filtered through the series of blast homology searches in the same manner as the coral SNPs described above. Finally, to confirm that the probes designed for the host and symbiont did not overlap, the final set of both groups were compared to each other using blastn with an e-value threshold of 1e−13. *Symbiodinium* non-polymorphic SNVs were identified from extracted genomic regions from the *S. ‘fitti’* scaffolds with the highest gene coverage for the *A. palmata* and *A. cervicornis* samples. After searching the non-polymorphic probes against each other using blastn (task = blastn, e-value = 1e−13), a random subset of 3,000 probes was selected.

In addition to the genotyping probes, we identified 12 SNPs in loci used to distinguish genera of Symbiodinaceae to capture potential background symbionts. The most common genera associated with tropical corals are *Symbiodinium*, *Breviolum*, *Cladocopium* and *Durisdinium* and can be distinguished by genetic markers. These loci include ribosomal (*internal transcribed spacer 2* and *nr28S*), mitochondrial (*COI* and *cob*), chloroplast (*cp23S* and *psbA*) and nuclear (*elongation factor 2*) markers using sequences from previously published studies^72–76^. Sequence accessions are provided in Supplemental Table S12. At least one representative sequence from each of the genera *Symbiodinium*, *Breviolum*, *Cladocopium* and *Durusdinium* for each locus was aligned with MUSCLE in Mega X^77^. SNPs were identified based on their ability to distinguish genera with enough conserved flanking sequence for probe design (Table S9).

### SNP validation by genotyping

After filtering, 34,783 acroporid SNPs (15,644 fixed, 10,429 population and 6,050 variable) and 2,661 symbiont SNPs were submitted for review by Affymetrix (Thermo Fisher, Santa Clarita, CA, USA). Final probe construction was completed by their bioinformatics team (Table S1). The final coral probe set was run through snpEff v4.3^78^ and the final algal probe set was compared to the respective GFF file for each *Symbiodinium* genome using bedtools intersect^70^ to determine genomic locations. The SNP density in bin sizes of 10,000 was extracted for all coral probes using VCFtools v0.1.15^68^.

Affymetrix optimized their current genotyping tools and pipeline to provide dual genotyping of the coral and symbiont in a single run. Five 96-well plates (Applied Biosystems Axiom Coral Genotyping Array—550962) were processed on the GeneTitan (Thermo Fisher, Santa Clarita, CA, USA). The raw data was analyzed using the Axiom ‘Best Practices Workflow’ (BPW) in the Axiom Analysis Suite software (Thermo Fisher, Santa Clarita, CA, USA) for each of the five runs separately for the coral and algal probe sets, with default quality filtering thresholds. Important thresholds that identify low sample quality include the dish quality, which is the signal of the non-polymorphic probes from one individual to the next, and call rate, which is the proportion of assigned genotypes for an individual out of all tested probes. The Bayesian clustering algorithm BRLMM-P^79^ was used to compute three posterior cluster locations (AA, AB, and BB) based on pre-positioned genotype cluster locations called priors. Genotype calls were made by identifying the intensity distribution, or cluster, each sample most likely belongs to with a confidence score (1 – posterior probability of the sample assignment to genotype cluster). In the case of the symbionts, because they are haploid, the algal genotyping probes were treated as mitochondrial probes with only homozygous AA or BB allele calls being valid. Five of the symbiont genera probes allowed for three clusters when multiple alleles were predicted to separate different genera (Table S9).

Following the analysis of the five plates, the performance of each probe was classified into six categories based on their separation of genotype clusters with SNPpolisher (Affymetrix, CA, USA) (Table S1). These categories include Poly High Resolution, Mono High Resolution, No Minor Hom, Call Rate Below Threshold, Other and Off-Target Variant. Probes that fell under ‘Poly High Resolution’ are those with resolution of three clusters (AA, AB and BB) with at least two sample having the minor allele. Probes that fell under ‘Mono High Resolution’ are those where all samples share the same allele possibly due to low minor allele frequency or sample selection on the plate. Finally, probes that fell under ‘No Minor Hom’ are those where no minor homozygous allele is observed, only AA and AB. These three categories make up the “best and recommended” probe set that was used in downstream analyses (Table S2, S3 and S10, and Fig. 2c).

### Standard tools for acroporid genotyping workflow

The general overview of the data conversion and genotype analysis steps are presented in Fig. S9a and code for new Galaxy tools can be found at (https://github.com/gregvonkuster/galaxy_tools/tree/master/tools/corals). Following the BPW, the genotypes were converted https://github.com/freeseek/gtc2vcf, filtered and combined with user-supplied metadata into a VCF (see Supplemental Methods), which are inputs for the *Coral Multilocus Genotype* tool executed through the R environment^80^. The VCF file was imported and converted into the genind format by the package vcfR v1.8.0^81^. The genind contains the individual genotypes that is then converted into a genclone format utilized by poppr v2.8.3 for clone identification^82,83^. A distance matrix is calculated within poppr using the Prevosti’s absolute genetic distance^84^, or the number of allelic differences between two individuals. From the distance matrix, known clone mates (ramets of the same genet) or replicate extractions from the same sample (Table S5) were compared to define a threshold for genet detection. This threshold encompasses technical (ie. missing alleles, genotyping error or DNA extraction differences) and biological (ie. somatic mutation) variation. The threshold was applied using *mlg.fitler* in poppr resulting in the assignment of samples to multi-locus genotype IDs, or genet IDs. Samples assigned to a genet ID with previously genotyped samples in the database took on the previous genet ID (ex. HG0000), whereas samples without matches to previously genotyped samples were assigned new genet IDs. The representative sample of the new genet ID was identified using the *clonecorrect* function in poppr. A series of tables were generated from the analysis and imported into the respective database tables using the *Update STAG Database* tool (Fig. S9a). This tool parses the metadata and genet information to append new records to the postgreSQL database (Fig. S10).

The genetic distance matrix was used to calculate a neighbor-joining tree with 100 bootstrap replicates using the *aboot* function in poppr. An identity-by-state analysis was performed using SNPRelate as previously described^46,85^. The representative sample for each genet ID (*n* = 193, excluding the genome samples, offspring of a Curacao cross with sample ID = SWSA, and plate 9SR22844), was used to identify populations with ADMIXTURE v1.3.0^86^ outside of the Galaxy portal. Plate 9SR22844 was excluded due to higher percentage of missing data (average 1.271 ± 0.581% out of 96 samples, Fig. S1e) and heterozygosity (average 14.163 ± 0.756% in *A. palmata* and 12.875 ± 1.020% in *A. cervicornis*, Fig. S2a) for the entire plate that contained only Puerto Rico samples compared to the Puerto Rico samples on plate P9SR10076 (average missing data of 0.501 ± 0.251% out of 73 samples and average heterozygosity of 13.801 ± 0.626% in *A. palmata* and 9.623 ± 0.446 in *A. cervicornis*, Fig. S1c). The exported VCF file from Galaxy was filtered for representative genets and loci were reduced after applying a minor allele threshold of 0.05 with VCFtools, and converted using PLINK v1.9^87^. First, all representative genets were analyzed with inferred population of K = 2 from 20 replicates with different random seeds to identify hybrids. Second, the two Caribbean species were split and populations of K ranging from 2 to 10 were run on each species separately over 20 replicates with different random seeds. In each iteration of ADMIXTURE, the replicates were combined and merged using the CLUMPAK server^88^.

Genotypes were extracted from the VCF file using the *extract.gt* tool in the vcfR package to determine the species of each sample. The nominally fixed probes were filtered further based on data from three plates where allele calls shared by less than 90% of all samples of a species were removed. Missing data was calculated for the full probe set and the fixed probe set. The percentage of heterozygous alleles (AB) and the percentage of homozygous alleles matching each species in the fixed probe set was calculated. A sample was identified as *A. palmata* or *A. cervicornis* if more than 85% of the fixed alleles match the respective species. Hybrid samples were identified as having 40% or greater heterozygosity.

### Galaxy CoralSNP analysis environment

The Galaxy Scientific Gateway called CoralSNP (https://coralsnp.science.psu.edu/galaxy) enables streamlined analysis of the Affymetrix genotype data described above to ultimately provide the user with a genet ID, converted raw genotype data, sample relatedness and hybrid status (Fig. 1). A baseline set of reports (https://coralsnp.science.psu.edu/reports) provides various views of the data, and additional reports will be added over time.

The straightforward process is shown in Figure S9b and described in additional detail in the Supplemental Methods. In brief, the user uploads their raw Affymetrix data files and metadata using the *Upload File* tool. Next, the user selects the appropriate files as inputs to the *Queue Genotype Workflow* tool (Fig. S9b), which validates the metadata (*Validate Affy Metadata* tool), executes the CoralSNP workflow (Fig. S9a) and updates a dataset that contains all previously genotyped samples as well as the STAG database (Fig. S10) with the samples in the current run (*Update STAG Database* tool).

It is imperative that the previously genotyped samples contained within this VCF file are synchronized with the previously genotyped sample records contained within the STAG database. The *Ensure Synced* tool confirms that the data contained within these two components is synchronized and creates backup copies of the VCF file and the database before updating either component. The Galaxy CoralSNP environment contains an independent tool named *Export All Sample Data*, which produces a tabular dataset consisting of all samples and associated metadata in the STAG database. This dataset can be saved locally for analysis within other environments. The dataset that contains all previously genotyped samples can also be downloaded from the Galaxy Data Library, providing more options for additional analyses outside of Galaxy.

All the code and configuration files needed for hosting a local Galaxy CoralSNP instance are available in GitHub, and the instructions for configuring the environment are here https://github.com/gregvonkuster/galaxy_tools/blob/master/galaxy/README. The CoralSNP workflow requires access to a dataset that contains all previously genotyped samples stored in a Galaxy Data Library (https://coralsnp.science.psu.edu/galaxy/library/list#folders/Fcba2ba6d6fdc5d84). The CoralSNP Science Gateway is hosted on a high-performance compute cluster environment managed by the Information Technology VM Hosting team at Pennsylvania State University.

### Symbiont genotyping: strain identification and background genera detection

The symbiont genotype data was analyzed in a similar manner to the coral data, but outside the Galaxy environment. Symbiont genotyping probes were identified from the BPW of all five plates after additional filtering to remove host contamination and low-resolution probes (*n* = 531, Table S3). The genotyping probes were subset using VCFtools and analyzed with a modified version of the *Coral Multilocus Genotype* tool. Notably, the ploidy was set to haploid. Because there was limited a priori information on the symbiont stains from microsatellite data, the distance threshold was set based on farthest and nearest threshold calculated by *cutoff_predictor* in poppr. Symbiont strains were given strain IDs in the format of SG0000.

For multiple vs. single strain detection from a single coral sample, five classification methods were used based on signal intensities of the filtered genotyping probes for samples assigned a strain ID. The intensities for each allele of each probe was extracted from the raw CEL file using Axiom Analysis Suite. Samples with prior symbiont genotyping from 12 to 13 microsatellites were used as the training set for all classification models where any sample with more than one allele per microsatellite marker was considered as containing multiple strains of *S. ‘fitti’* (*n* = 17 samples with multiple strains and *n* = 11 samples with a single strain). The remaining samples were the test set (*n* = 265). The two data sets were centered and scaled prior to analysis. The five classification tests included supervised learning models such as linear discriminant analysis (LDA) (MASS v7.3-51.4 R package^89^), decision tree (rpart v4.1-15 R package^90^ and rpart.plot v3.0.8 R package^91^), random forest (caret v6.0–84 R package^92^), naïve Bayes (caret v6.0–84 R package^92^), and semi-supervised learning model using k nearest-neighbor masking 30% of the training data (SSC v2.0.0 R package^93^). All tests, except for the LDA, were resampled three times with tenfold cross-validation to evaluate model fit. The results of the five tests are presented as the percent of multiple strain assignment for each genotyped sample.

The background genera were assigned based on the fit of three of the classification tests above: LDA, decision tree and random forest. All samples and probes were first visualized in the Axiom Analysis Suite software to identify patterns in samples with known background symbiont populations (*A. cervicornis* with *Cladocopium: n* = *2*^94^, *A. cervicornis* with *Durusdinium: n* = *2*, Pacific acroporids with *Cladocopium: n* = 20 and *A. muricata* with *Durusdinium: n* = 5^65^). Probes were filtered based on their recommended status (Table 1) and assignment of known samples above. A preliminary assignment of symbionts to genera was made for each sample based on their cluster patterns. The signal intensity for the genera probes (*n* = 18) was extracted for all samples regardless of their genotype status using the Axiom Analysis Suite. The data was split into 80% for training and 20% for testing. Cross-validation was performed on the decision tree and random forest models as described above.

## Data availability

The Galaxy CoralSNP analysis environment and database reports are available at https://coralsnp.science.psu.edu/reports. The metadata template is available at https://baumslab.org/research/data/. A tutorial for executing the analysis workflow is available https://protocols.io/view/tutorial-to-use-the-galaxy-coral-snp-stagdb-workfl-beqcjdsw.html. Protocols are available for how to sample corals for genotyping (10.17504/protocols.io.bec8jazw) and how to extract DNA (10.17504/protocols.io.bgjqjumw). The code for the new tools developed for this study are available at https://github.com/gregvonkuster/galaxy_tools/tree/master/tools/corals and https://github.com/gregvonkuster/galaxy_tools/tree/master/galaxy. Sequences for the genome samples are available on NCBI under SRA project SRP149363. The coral probe annotation is provided in Supplemental File 1 and the symbiont probe annotation is provided in Supplemental File 2. The Applied Biosystems Axiom Coral genotyping array is available in 96 (#550962) and 384 (#550961) sample format.

## Supplementary information

Supplementary Information 1.

Supplementary Information 2.

Supplementary Information 3.

Supplementary Information 4.

Supplementary Information 5.

## References

[1] Altshuler D (2000). An SNP map of the human genome generated by reduced representation shotgun sequencing. Nature.

[2] Ali OA (2016). RAD capture (Rapture): flexible and efficient sequence-based genotyping. Genetics.

[3] Hoffberg SL (2016). RADcap: sequence capture of dual-digest RADseq libraries with identifiable duplicates and reduced missing data. Mol. Ecol. Resour..

[4] Franchini P, Monné Parera D, Kautt AF, Meyer A (2017). quaddRAD: a new high-multiplexing and PCR duplicate removal ddRAD protocol produces novel evolutionary insights in a nonradiating cichlid lineage. Mol. Ecol..

[5] Darrier B (2019). A comparison of mainstream genotyping platforms for the evaluation and use of barley genetic resources. Front. Plant Sci..

[6] Palti Y (2015). The development and characterization of a 57 K single nucleotide polymorphism array for rainbow trout. Mol. Ecol. Resour..

[7] Moragues M (2010). Effects of ascertainment bias and marker number on estimations of barley diversity from high-throughput SNP genotype data. Theor. Appl. Genet..

[8] Malomane DK (2018). Efficiency of different strategies to mitigate ascertainment bias when using SNP panels in diversity studies. BMC Genomics.

[9] Lachance J, Tishkoff SA (2013). SNP ascertainment bias in population genetic analyses: why it is important, and how to correct it. BioEssays.

[10] Afgan E (2018). The Galaxy platform for accessible, reproducible and collaborative biomedical analyses: 2018 update. Nucleic Acids Res..

[11] Whitaker K (2006). Genetic evidence for mixed modes of reproduction in the coral *Pocillopora damicornis* and its effect on population structure. Mar. Ecol. Prog. Ser..

[12] Miller KJ, Ayre DJ (2004). The role of sexual and asexual reproduction in structuring high latitude populations of the reef coral *Pocillopora damicornis*. Heredity.

[13] Stoddart JA (1983). Asexual production of planulae in the coral *Pocillopora damicornis*. Mar. Biol..

[14] Ayre DJ, Hughes TP (2000). Genotypic diversity and gene flow in brooding and spawning corals along the Great Barrier Reef, Australia. Evolution.

[15] Adjeroud M, Tsuchiya M (1999). Genetic variation and clonal structure in the scleractinian coral *Pocillopora damicornis* in the Ryukyu Archipelago, southern Japan. Mar. Biol..

[16] Foster NL, Baums IB, Mumby PJ (2007). Sexual vs. asexual reproduction in an ecosystem engineer: the massive coral *Montastraea annularis*. J. Anim. Ecol..

[17] Neigel JE, Avise JC (1983). Clonal diversity and population structure in a reef-building coral, *Acropora cervicornis*: self-recognition analysis and demographic interpretation. Evolution.

[18] Baums IB, Miller MW, Hellberg ME (2006). Geographic variation in clonal structure in a reef building Caribbean coral, *Acropora palmata*. Ecol. Monogr..

[19] Pinzón J, Reyes-Bonilla H, Baums I, LaJeunesse T (2012). Contrasting clonal structure among *Pocillopora* (Scleractinia) communities at two environmentally distinct sites in the Gulf of California. Coral Reefs.

[20] Parkinson JE, Baums IB (2014). The extended phenotypes of marine symbioses: ecological and evolutionary consequences of intraspecific genetic diversity in coral-algal associations. Front. Microbiol..

[21] Polato NR, Altman NS, Baums IB (2013). Variation in the transcriptional response of threatened coral larvae to elevated temperatures. Mol. Ecol..

[22] Baums I (2013). Genotypic variation influences reproductive success and thermal stress tolerance in the reef building coral, *Acropora palmata*. Coral Reefs.

[23] Randall CJ, Szmant AM (2009). Elevated temperature affects development, survivorship, and settlement of the Elkhorn coral, *Acropora palmata* (Lamarck 1816). Biol. Bull..

[24] Meyer E (2009). Genetic variation in responses to a settlement cue and elevated temperature in the reef-building coral *Acropora millepora*. Mar. Ecol. Prog. Ser..

[25] Baums IB, Hughes CR, Hellberg MH (2005). Mendelian microsatellite loci for the Caribbean coral *Acropora palmata*. Mar. Ecol. Prog. Ser..

[26] Fogarty ND, Vollmer SV, Levitan DR (2012). Weak Prezygotic isolating mechanisms in threatened Caribbean *Acropora* corals. PLoS ONE.

[27] Baums IB (2019). Considerations for maximizing the adaptive potential of restored coral populations in the western Atlantic. Ecol. Appl..

[28] Muscatine L, Cernichiari E (1969). Assimilation of photosynthetic products of zooxanthellae by a reef coral. Biol. Bull..

[29] Davies PS (1991). Effect of daylight variations on the energy budgets of shallow-water corals. Mar. Biol..

[30] Santos SR, Coffroth MA (2003). Molecular genetic evidence that dinoflagellates belonging to the genus *Symbiodinium* Freudenthal are haploid. Biol. Bull..

[31] Pettay DT (2007). LaJeunesse TC (2007) Microsatellites from clade B *Symbiodinium* spp. specialized for Caribbean corals in the genus *Madracis*. Mol. Ecol. Notes.

[32] Pettay DT, LaJeunesse TC (2009). Microsatellite loci for assessing genetic diversity, dispersal and clonality of coral symbionts in ‘stress-tolerant’ clade D *Symbiodinium*. Mol. Ecol. Resour..

[33] Pinzón JH, Devlin-Durante MK, Weber MX, Baums IB, LaJeunesse TC (2011). Microsatellite loci for *Symbiodinium* A3 (*S. fitti*) a common algal symbiont among Caribbean Acropora (stony corals) and Indo-Pacific giant clams (Tr*i*dacna). Conserv. Genet. Resour..

[34] Baums IB, Devlin-Durante MK, LaJeunesse TC (2014). New insights into the dynamics between reef corals and their associated dinoflagellate endosymbionts from population genetic studies. Mol. Ecol..

[35] Wham DC, Pettay DT, LaJeunesse TC (2011). Microsatellite loci for the host-generalist “zooxanthella” *Symbiodinium trenchi* and other Clade D *Symbiodinium*. Conserv. Genet. Resour..

[36] Grupstra CG (2017). Evidence for coral range expansion accompanied by reduced diversity of *Symbiodinium* genotypes. Coral Reefs.

[37] Chan AN, Lewis CL, Neely KL, Baums IB (2019). Fallen pillars: the past, present, and future population dynamics of a rare, specialist coral-algal symbiosis. Front. Mar. Sci..

[38] Andras JP, Kirk NL, Coffroth MA, Harvell CD (2009). Isolation and characterization of microsatellite loci in *Symbiodinium* B1/B184, the dinoflagellate symbiont of the Caribbean sea fan coral, *Gorgonia ventalina*. Mol. Ecol. Resour..

[39] Veron JEN (2000). Corals of the World.

[40] Wallace CC (1999). Staghorn Corals of the World: A Revision of the Coral Genus *Acropora* (Scleractinia; Astrocoeniina; Acroporidae) Worldwide, with Emphasis on Morphology, Phylogeny and Biogeography.

[41] van Oppen MJH, Willis BL, van Vugt JA, Miller DJ (2000). Examination of species boundaries in the *Acropora cervicornis* group (Scleractinia, Cnidaria) using nuclear DNA sequence analyses. Mol. Ecol..

[42] Vollmer SV, Palumbi SR (2002). Hybridization and the evolution of reef coral diversity. Science.

[43] de Lamarck JBPA (1816). Histoire Naturelle des Animaux sans Vertebres.

[44] Li H (2011). A statistical framework for SNP calling, mutation discovery, association mapping and population genetical parameter estimation from sequencing data. Bioinformatics.

[45] 45Garrison, E. & Marth, G. Haplotype-based variant detection from short-read sequencing. arXiv:1207.3907 (2012).

[46] Kitchen SA (2019). Genomic variants among threatened *Acropora* corals. G3: Genes Genomes Genet..

[47] Liew YJ, Aranda M, Voolstra CR (2016). Reefgenomics.org—a repository for marine genomics data. Database.

[48] Fuller ZL (2019). Population genetics of the coral *Acropora millepora*: towards a genomic predictor of bleaching. bioRxiv.

[49] 49Hong, H. *et al.* in *BMC Bioinformatics.* (BioMed Central).

[50] Hong H (2012). Technical reproducibility of genotyping SNP arrays used in genome-wide association studies. PLoS ONE.

[51] Lee YG (2015). Development, validation and genetic analysis of a large soybean SNP genotyping array. Plant J..

[52] Marrano A (2019). A new genomic tool for walnut (*Juglans regia* L.): development and validation of the high-density Axiom™ *J.**regia* 700K SNP genotyping array. Plant Biotechnol. J..

[53] Baums IB, Johnson ME, Devlin-Durante MK, Miller MW (2010). Host population genetic structure and zooxanthellae diversity of two reef-building coral species along the Florida Reef Tract and wider Caribbean. Coral Reefs.

[54] Hemond EM, Vollmer SV (2010). Genetic diversity and connectivity in the threatened Staghorn coral (*Acropora cervicornis*) in Florida. PLoS ONE.

[55] Vollmer SV, Palumbi SR (2007). Restricted gene flow in the Caribbean staghorn coral *Acropora cervicomis*: Implications for the recovery of endangered reefs. J. Hered..

[56] Drury C (2016). Genomic variation among populations of threatened coral: *Acropora cervicornis*. BMC Genomics.

[57] Porto-Hannes I (2014). Population structure of the corals *Orbicella faveolata* and *Acropora palmata* in the Mesoamerican Barrier Reef System with comparisons over Caribbean basin-wide spatial scale. Mar. Biol..

[58] Baums IB, Miller MW, Hellberg ME (2005). Regionally isolated populations of an imperiled Caribbean coral, *Acropora palmata*. Mol. Ecol..

[59] Devlin-Durante MK, Baums IB (2017). Genome-wide survey of single-nucleotide polymorphisms reveals fine-scale population structure and signs of selection in the threatened Caribbean elkhorn coral, *Acropora palmata*. PeerJ.

[60] Palumbi SR, Vollmer S, Romano S, Oliver T, Ladner J (2012). The role of genes in understanding the evolutionary ecology of reef building corals. Evol. Ecol..

[61] Miller DJ, Van Oppen MJH (2003). A 'fair go' for coral hybridization. Mol. Ecol..

[62] Japaud A, Bouchon C, Magalon H, Fauvelot C (2019). Geographic distances and ocean currents influence Caribbean *Acropora palmata* population connectivity in the Lesser Antilles. Conserv. Genet..

[63] Liu H (2018). *Symbiodinium* genomes reveal adaptive evolution of functions related to coral-dinoflagellate symbiosis. Commun. Biol..

[64] Thornhill DJ, LaJeunesse TC, Kemp DW, Fitt WK, Schmidt GW (2006). Multi-year, seasonal genotypic surveys of coral-algal symbioses reveal prevalent stability or post-bleaching reversion. Mar. Biol..

[65] Hoadley KD (2019). Host–symbiont combinations dictate the photo-physiological response of reef-building corals to thermal stress. Sci. Rep..

[66] Fukami H (2017). Geographic differences in species boundaries among members of the *Montastraea annularis* complex based on molecular and morphological markers. Evolution.

[67] Hatta M (1999). Reproductive and genetic evidence for a reticulate evolutionary history of mass-spawning corals. Mol. Biol. Evol..

[68] Danecek P (2011). The variant call format and VCFtools. Bioinformatics.

[69] Shinzato C (2011). Using the *Acropora digitifera* genome to understand coral responses to environmental change. Nature.

[70] Quinlan AR, Hall IM (2010). BEDTools: a flexible suite of utilities for comparing genomic features. Bioinformatics.

[71] Shoguchi E (2018). Two divergent *Symbiodinium* genomes reveal conservation of a gene cluster for sunscreen biosynthesis and recently lost genes. BMC Genomics.

[72] Takishita K, Ishikura M, Koike K, Maruyama T (2003). Comparison of phylogenies based on nuclear-encoded SSU rDNA and plastid-encoded psbA in the symbiotic dinoflagellate genus *Symbiodinium*. Phycologia.

[73] Pochon X, Putnam HM, Burki F, Gates RD (2012). Identifying and characterizing alternative molecular markers for the symbiotic and free-living dinoflagellate genus *Symbiodinium*. PLoS ONE.

[74] Arif C (2014). Assessing *Symbiodinium* diversity in scleractinian corals via next-generation sequencing-based genotyping of the ITS2 rDNA region. Mol. Ecol..

[75] LaJeunesse TC (2002). Diversity and community structure of symbiotic dinoflagellates from Caribbean coral reefs. Mar. Biol..

[76] LaJeunesse TC (2001). Investigating the biodiversity, ecology, and phylogeny of endosymbiotic dinoflagellates in the genus *Symbiodinium* using the ITS region: in search of a “species” level marker. J. Phycol..

[77] Kumar S, Stecher G, Li M, Knyaz C, Tamura K (2018). MEGA X: molecular evolutionary genetics analysis across computing platforms. Mol. Biol. Evol..

[78] Cingolani P (2012). A program for annotating and predicting the effects of single nucleotide polymorphisms, SnpEff: SNPs in the genome of *Drosophila melanogaster* strain w1118; iso-2; iso-3. Fly.

[79] Affymetrix. (Affymetrix, 2007).

[80] R: a language and environment for statistical computing [Online] (R Foundation for Statistical Computing, Vienna, 2017).

[81] Knaus BJ, Grünwald NJ (2017). vcfr: a package to manipulate and visualize variant call format data in R. Mol. Ecol. Resour..

[82] Kamvar ZN, Brooks JC, Grünwald NJ (2015). Novel R tools for analysis of genome-wide population genetic data with emphasis on clonality. Front. Genet..

[83] Kamvar ZN, Tabima JF, Grünwald NJ (2014). Poppr: an R package for genetic analysis of populations with clonal, partially clonal, and/or sexual reproduction. PeerJ.

[84] Prevosti A, Ocana J, Alonso G (1975). Distances between populations of *Drosophila subobscura*, based on chromosome arrangement frequencies. Theor. Appl. Genet..

[85] Zheng X (2012). A high-performance computing toolset for relatedness and principal component analysis of SNP data. Bioinformatics.

[86] Alexander DH, Novembre J, Lange K (2009). Fast model-based estimation of ancestry in unrelated individuals. Genome Res..

[87] Chang CC (2015). Second-generation PLINK: rising to the challenge of larger and richer datasets. Gigascience.

[88] Kopelman NM, Mayzel J, Jakobsson M, Rosenberg NA, Mayrose I (2015). Clumpak: a program for identifying clustering modes and packaging population structure inferences across K. Mol. Ecol. Resour..

[89] Venables W, Ripley B (2002). Modern Applied Statistics with S.

[90] Therneau, T. & Atkinson, B. *rpart: Recursive Partitioning and Regression Trees*. R package version 4.1-15 (2019). https://CRAN.R-project.org/package=rpart.

[91] Milborrow, S. *rpart.plot: Plot ‘rpart’ Models: An Enhanced Version of ‘plot.rpart*’. R package version 3.0.8 (2019). https://CRAN.R-project.org/package=rpart.plot.

[92] Kuhn M (2008). Building predictive models in R using the caret package. J. Stat. Softw..

[93] González, M., Rosado-Falcón, O. & Rodríguez, J. D. *ssc: Semi-Supervised Classification Methods*. R package version 2.1-0 (2019). https://CRAN.R-project.org/package=ssc.

[94] Lirman D (2014). Growth dynamics of the threatened Caribbean staghorn coral *Acropora cervicornis:* influence of host genotype, symbiont identity, colony size, and environmental setting. PLoS ONE.

