# Supplementary material for: STAGdb: a 30K SNP genotyping array and Science Gateway for Acropora corals and their dinoflagellate symbionts
Source: Sci Rep. 2020 Jul 27;10:12488. doi: 10.1038/s41598-020-69101-z (PMC7385180; doi:10.1038/s41598-020-69101-z)
Supplement: Supplementary file 3 — Supplementary Information 3. [file 41598_2020_69101_MOESM3_ESM.docx]

# Supplemental file for:

# STAGdb: a 30K SNP genotyping array and Science Gateway for *Acropora* corals and their dinoflagellate symbionts

S. A. Kitchen, G. Von Kuster, K.L.Vasquez Kuntz, H.G. Reich, W. Miller, S. Griffin, N.D. Fogarty and I.B. Baums

**Supplemental Methods**

## Coral collection and DNA extraction

Initially samples were selected from an archival tissue collection to address technical concerns regarding: dual genotyping with enrichment of host or symbiont DNA, tissue type (sperm, larvae and adult), clone identification (multiple ramets per genet), reproducibility between labs (Baums lab and Fogarty lab), and difference in tissue preservatives (flash frozen, non-denatured 95-100% ethanol, CHAOS buffer and DMSO). We also included several *Acropora* samples from the Pacific including *A. muricata*, *A. millepora* and *A. digitifera* (generously donated by Drs. Todd LaJeunesse, Zachary Fuller, Mikhail Matz and Stephen Palumbi). The remaining samples include archival samples collected from various locations across the geographic distribution of *A. palmata* and *A. cervicornis* and their hybrid, *A. prolifera*. Sample information can be found in Supplemental Table S4.

DNA extraction methods are available in detail on supporting protocols.io repository (<https://www.protocols.io/view/stagdb-a-30k-snp-genotyping-array-and-science-gate-bcwtixen/abstract>). High molecular weight DNA from concentrated coral sperm was extracted using the illustra Nucleon Phytopure kit (GE Healthcare Life Science, Pittsburgh, PA) following the manufacturer’s instructions and eluted in nuclease-free water. For coral larvae, an individual larva was incubated in 12 µl of lysis solution (10.8 µl Buffer TL (Omega BioTek, Norcross, GA), 1 µl of OB Protease Solution (Omega BioTek) and 0.2 µl of RNAse A (100 mg/ml)) for 20 min at 55 °C. An additional 38 µl of Buffer TL was added to each sample followed by 50 µl of phenol/chloroform/isoamyl alcohol solution (25:24:1) and gently rocked for 2 min. The samples were centrifuged for 10 min at 10,000 rpm. To the aqueous phase, 50 µl of chloroform:isoamyl alcohol (24:1) was added and mixed with gentle rocking for 2 min. The aqueous phase was recovered after 5 min centrifugation at 10,000 rpm. The DNA was precipitated with 1.5x volume of room-temperature isopropanol, 1/10 volume of 3M sodium acetate (pH=5.2) and 1 µl of glycogen (5 mg/ml) for 10 min at room temperature followed by centrifugation at 15,000 rpm for 20 min and two rounds of washes with 70% ethanol. The pellets were resuspended in 20 µl of low TE buffer (10 mM Tris-HCl and 0.1 mM EDTA). In the Baums lab, DNA from adult coral tissue was extracted using the Qiagen DNeasy kit (Qiagen, Valencia, CA) following the manufacturer’s protocol and eluted in 100 µl of nuclease-free water or low TE buffer. A subset of adult tissue was extracted separately by the Fogarty lab. These samples were either preserved directly in CHAOS DNA extraction buffer^1^ or ethanol and the DNA was isolated using a magnetic bead protocol^2^. These methods are referred to as a “mixed” extraction because both coral and symbiont DNA is recovered, but in unknown proportions. For enriched symbiont DNA from the coral tissue, we isolated the symbionts using a modification of Wayne’s method^3^ described by Bongaerts, et al. ^4^. Briefly, ~3-4 coral calyces were placed in 600 µl of nuclease-free water and vortexed on maximum speed for 1 min to remove the tissue from the coral skeleton. The skeleton pieces were removed and the supernatant with the coral tissue was centrifuged for 3 min at 2,500 rpm. The supernatant was removed leaving a pellet of symbiont cells. Glass beads were added to the symbiont pellet and vortexed on high for 1 min to disrupt the cell membrane. High-molecular weight DNA quality was assessed using gel electrophoresis, and yield quantified using either NanoDrop 2000 (Thermo Scientific) or PicoGreen assay (Invitrogen) for sperm and adult extractions and a Qubit fluorometer dsDNA Broad Range kit (Invitrogen) for the larval extractions.

## Genotyping workflow and Galaxy CoralSNP analysis environment

### Pre-processing SNP data

The resulting genotype files from the BPW were first converted into the variant call format (VCF) using the bcftools plugin affy2vcf (<https://github.com/freeseek/gtc2vcf>). The VCF files were sorted and merged using bcftools (Fig. S9a). The coral genotyping probes recommended from the BPW with the first plate were subset from the VCF of previous genome samples using VCFtools. These SNPs were marked in the INFO field that was then used for filtering after the array data was merged with the genome samples (Fig. S9a, “Select” step). The Affymetrix IDs and the order of the samples were extracted from the filtered VCF using the *Affy Ids for Genotyping* tool and combined with the sample attributes from the BPW and population information in the user-supplied metadata into a new text file with the *Genotype Population Info* tool (Fig. S9a). Additional population information was appended to the file from the previously genotyped samples in the database.

### Details of Galaxy CoralSNP Analysis

The Galaxy Scientific Gateway called CoralSNP (<https://coralsnp.science.psu.edu>/galaxy) provide the user with a genet ID, converted raw genotype data, sample relatedness and hybrid status through an automated workflow (Fig. 1). A baseline set of reports (<https://coralsnp.science.psu.edu/reports>) provides various views of the data.

To begin, a sample metadata file is created by the user using a template form (<http://baumslab.org/research/data/>). The metadata file contains a field where the user can choose when their data becomes publicly available, allowing up to a year hold. The user then uploads their raw Affymetrix data files and metadata into the Galaxy CoralSNP environment using the *Upload File* tool in the Galaxy tool panel. Next, the user selects the eight data files as inputs to the *Queue Genotype Workflow* tool (Fig. S9b): the user metadata file and the Affymetrix sample attributes, annotation, summary, snp-posteriors, report, confidences and calls files. Within the *Queue Genotype Workflow*, it executes a series of subtools to validate the metadata (*Validate Affy Metadata* tool), execute the CoralSNP workflow (Fig. S9a) and update a dataset that contains all previously genotyped samples as well as the STAG database (Fig. S10) with the samples in the current run (*Update STAG Database* tool). Once the tool is executed, the user can simply wait for the CoralSNP analysis pipeline to finish in the right panel.

The *Queue Genotype Workflow* tool shields the complexity of the analysis from the user and performs its functions via the Galaxy REST API. *Affy_ids_for_genotyping* tool extracts information from a VCF files that contains Affymetrix identifiers and produces a file that contains a subset of the identifiers combined with additional data to generate the genotype population information for use as input to the *Coral Multilocus Genotype* tool. From there, the *Genotype_population_info* tool generates the genotype population information file for use as input to the *Coral Multilocus Genotype* tool. The *Coral_multilocus_genotype* tool renders the unique combination of alleles for two or more loci for each individual using the steps described in Standard Tools for Acroporid Genotyping Workflow methods section. After the genotypes are computed for the new samples, the *Update_stag_database* tool updates the postgreSQL database tables from a dataset collection where each item in the collection is a tabular file that will be parsed to insert rows into a table defined by the name of the file. The code for these tools is available in GitHub at <https://github.com/gregvonkuster/galaxy_tools/tree/master/tools/corals>.

The CoralSNP workflow requires access to a dataset that contains all previously genotyped samples, so the tool imports this dataset into the user’s current Galaxy history from a Galaxy Data Library (https://coralsnp.science.psu.edu/galaxy/library/list#folders/Fcba2ba6d6fdc5d84). It is imperative that the previously genotyped samples contained within this VCF file are synchronized with the previously genotyped sample records contained within the STAG database. The *Ensure Synced* tool confirms that the data contained within these two components is synchronized before proceeding. The tool makes backup copies of the VCF file and the database before updating either component. Since both components are updated, multiple simultaneous analyses cannot be performed. The *Queue genotype workflow* tool handles this by ensuring that multiple simultaneous executions are handled serially. This is done by polling the status of the first execution until it has completed. Additional simultaneous executions are queued in the order in which they were submitted. If an analysis ends in an error with either the VCF file or the database updated so that they are no longer in sync, the backup copy of the appropriate component can be used to replace the problematic one in preparation for the next run.

The Galaxy CoralSNP environment contains an independent tool named *Export All Sample Data*, which produces a tabular dataset consisting of all samples and associated metadata in the STAG database. This dataset can be saved locally for analysis within other environments. The dataset that contains all previously genotyped samples can also be downloaded from the Galaxy Data Library, providing more options for additional analyses outside of Galaxy.

All the code and configuration files needed for hosting a local Galaxy CoralSNP instance are available in GitHub, and the instructions for configuring the environment are here <https://github.com/gregvonkuster/galaxy_tools/blob/master/galaxy/README>. The CoralSNP Science Gateway is hosted on a high-performance compute cluster environment managed by the Information Technology VM Hosting team at Pennsylvania State University.

**Table S1. Number of recommended probes for each taxon from five independent runs.**

|  |  | **Caribbean *Acropora*** | | | | **Symbiont** | | | |
| --- | --- | --- | --- | --- | --- | --- | --- | --- | --- |
| **Plate Number** | **Probe Classification** | **Fixed** | **Population** | **Variable** | **Total** | **Fixed** | **Population** | **Genera** | **Total** |
| **All probes** |  | **25,889** | **17,803** | **9,887** | **53,579** | **3,663** | **304** | **54** | **4,021** |
| **Plate 1** | **Recommended** | **9,919** | **6,455** | **3,342** | **19,716** | **2,176** | **109** | **18** | **2,303** |
|  | polyHigh | 9,663 | 5,855 | 2,515 | 18,033 | 2,093 | 68 | 0 | 2,161 |
|  | MonoHigh | 181 | 173 | 524 | 878 | 83 | 41 | 14 | 138 |
|  | noMinor | 75 | 427 | 303 | 805 | 0 | 0 | 0 | 0 |
| **Plate 2** | **Recommended** | **10,199** | **6,701** | **3,445** | **20,345** | **2,210** | **100** | **15** | **2,325** |
|  | polyHigh | 9,987 | 6,381 | 2,759 | 19,127 | 1,792 | 68 | 1 | 1,861 |
|  | MonoHigh | 165 | 164 | 572 | 901 | 418 | 32 | 12 | 462 |
|  | noMinor | 47 | 156 | 114 | 317 | 0 | 0 | 2 | 2 |
| **Plate 3** | **Recommended** | **12,299** | **7,823** | **4,137** | **24,259** | **2,189** | **109** | **17** | **2,315** |
|  | polyHigh | 11,916 | 6,918 | 3,249 | 22,083 | 261 | 54 | 0 | 315 |
|  | MonoHigh | 195 | 189 | 681 | 1,065 | 1,928 | 55 | 14 | 1,997 |
|  | noMinor | 188 | 716 | 207 | 1,111 | 0 | 0 | 3 | 3 |
| **Plate 4** | **Recommended** | **10,171** | **7,178** | **3.814** | **21,163** | **2,247** | **107** | **16** | **2,370** |
|  | polyHigh | 8,989 | 5,783 | 2,827 | 17.599 | 223 | 48 | 0 | 271 |
|  | MonoHigh | 435 | 593 | 774 | 1,802 | 2,024 | 59 | 16 | 2,099 |
|  | noMinor | 746 | 802 | 213 | 1,761 | 0 | 0 | 0 | 0 |
| **Plate 5** | **Recommended** | **11,093** | **7,620** | **4,038** | **22.751** | **2,215** | **113** | **17** | **2,345** |
|  | polyHigh | 8,748 | 5,662 | 2,498 | 16,908 | 621 | 37 | 1 | 659 |
|  | MonoHigh | 459 | 580 | 821 | 1,860 | 1,594 | 76 | 16 | 1,686 |
|  | noMinor | 1,886 | 1,378 | 719 | 3.983 | 0 | 0 | 0 | 0 |

**Table S4. Success rate after quality-filtering of Caribbean acroporid and symbiont samples.** To the left of the slash are those samples that passed the default quality filtering of the Best Practices Workflow (BPW) and to the right is the total number of processed samples.

|  | Plate 1 | | Plate 2 | | Plate 3 | | Plate 4 | | Plate 5 | |
| --- | --- | --- | --- | --- | --- | --- | --- | --- | --- | --- |
|  | Coral | Symbiont | Coral | Symbiont | Coral | Symbiont | Coral | Symbiont | Coral | Symbiont |
| All samples | 90/95 | 81/90* | 92/96 | 78/95* | 93/95 | 29/84 | 72/96 | 23/72* | 90/96 | 70/96* |
| *Mixed extractions* | 81/84 | 76/89* | 91/95 | 78/95* | 83/83 | 29/83 | 72/72 | 23/72* | 90/96 | 70/96* |
| *Symbiont-enriched extractions* | 3/5 | 5/5 | -- | -- | -- | -- | -- | -- | -- | -- |
| *Sperm* | 1/1 | -- | 1/1 | -- | -- | -- | -- | -- | -- | -- |
| *Larvae* | 5/5 | -- | -- | -- | 10/12 | -- | -- | -- | -- | -- |
| *Symbiont culture* | -- | 0/1* | -- | -- | -- | 0/1* | -- | -- | -- | -- |
| *Indo-Pacific acroporid* | 0/1* | -- | -- | -- | -- | -- | 0/24* | -- | -- | -- |

* Failed on dish quality (signal observed from non-polymorphic loci) – indication of different coral/symbiont species or background symbionts.

**Table S6. Genetic distance between replicate DNA extractions and ramets of Sand Island samples.** Distances of DNA extractions originating from the same tissue sample are colored the same whereas distances between ramets are not colored.

| Sample ID | SI-1.1 | SI-1.2 | SI-1.3 | SI-10.1 | SI-10.2 | SI-10.3 | SI-12.1 | SI-12.2 | 11956 |
| --- | --- | --- | --- | --- | --- | --- | --- | --- | --- |
| SI-1.1 | 0 |  |  |  |  |  |  |  |  |
| SI-1.2 | 0.0041 | 0 |  |  |  |  |  |  |  |
| SI-1.3 | 0.0051 | 0.0049 | 0 |  |  |  |  |  |  |
| SI-10.1 | 0.0034 | 0.0047 | 0.0059 | 0 |  |  |  |  |  |
| SI-10.2 | 0.0071 | 0.0068 | 0.0061 | 0.0068 | 0 |  |  |  |  |
| SI-10.3 | 0.0020 | 0.0043 | 0.0050 | 0.0028 | 0.0063 | 0 |  |  |  |
| SI-12.1 | 0.0054 | 0.0054 | 0.0046 | 0.0049 | 0.0048 | 0.0042 | 0 |  |  |
| SI-12.2 | 0.0082 | 0.0067 | 0.0085 | 0.0076 | 0.0077 | 0.0078 | 0.0069 | 0 |  |
| 11956 | 0.0065 | 0.0093 | 0.0092 | 0.0071 | 0.0109 | 0.0060 | 0.0090 | 0.0123 | 0 |

**Table S8. Reproducibility of genet identification between laboratories.**

| User ID | Msat Genet ID | Preservative | Extraction Method | Lab | DNA concentration (ng/ul) | SNP Genet ID | Missing Data (%) | Heterozygosity (%) | A. cervicornis | A. palmata | Genetic Distance |
| --- | --- | --- | --- | --- | --- | --- | --- | --- | --- | --- | --- |
| 13935 | C1522 | Ethanol | Qiagen DNeasy | Baums | 34.71 | HG0136 | 1.44 | 1.06 | 97.66 | 0.22 | 0.020 |
| 13935_NF | C1522 | Ethanol | CHAOS | Fogarty | 0.064 | HG0136 | 1.96 | 6.01 | 91 | 0.21 |  |
| 3845 | C1548 | Ethanol | Qiagen DNeasy | Baums | 22.76 | HG0003 | 0.36 | 0.57 | 98.81 | 0.3 | 0.018 |
| 3845_NF | C1548 | Ethanol | CHAOS | Fogarty | 1.894 | HG0003 | 1.49 | 5.71 | 91.96 | 0.22 |  |
| 13716 | C1639 | Ethanol | Qiagen DNeasy | Baums | 16.97 | HG0006 | 1.23 | 2.04 | 96.51 | 0.29 | 0.027 |
| 126_NF | C1639 | CHAOS | CHAOS | Fogarty | 4.669 | HG0006 | 2.28 | 7.45 | 88.75 | 0.22 |  |
| 13829 | C1643 | Ethanol | Qiagen DNeasy | Baums | 9.27 | HG0029 | 0.63 | 1.54 | 97.51 | 0.29 | 0.019 |
| 13829_NF | C1643 | Ethanol | CHAOS | Fogarty | 8.975 | HG0029 | 1.41 | 5.99 | 91.77 | 0.29 |  |
| 13839 | C1645 | Ethanol | Qiagen DNeasy | Baums | 19.21 | HG0059 | 0.2 | 0.79 | 98.32 | 0.75 | 0.017 |
| 13839_NF | C1645 | Ethanol | CHAOS | Fogarty | 1.762 | HG0059 | 1.37 | 5.8 | 91.59 | 0.71 |  |
| 13923 | C1652 | Ethanol | Qiagen DNeasy | Baums | 3.41 | HG0153 | 1.36 | 0.53 | 98.57 | 0.29 | 0.027 |
| 13923_NF | C1652 | Ethanol | CHAOS | Fogarty | 6.951 | HG0153 | 2.55 | 7.69 | 89.01 | 0.33 |  |
| 13756 | C1343 | Ethanol | Qiagen DNeasy | Baums | 2.504 | HG0144 | 0.96 | 0.73 | 98.43 | 0.3 | 0.019 |
| 166_NF | C1343 | CHAOS | CHAOS | Fogarty | 4.951 | HG0144 | 1.78 | 6.28 | 90.89 | 0.29 |  |
| 1151 | P1020 | Ethanol | Qiagen DNeasy | Baums | 22.16 | HG0171 | 1.08 | 1.3 | 0.3 | 97.59 | 0.012 |
| 1151_NF | P1020 | Ethanol | CHAOS | Fogarty | 8.154 | HG0171 | 1.42 | 3.32 | 0.3 | 94.64 |  |

**Table S9. Symbiont genera probes.**

| Affy SNP ID | Probe Set ID | Gene ID | Probe | Allele for each Genus* |
| --- | --- | --- | --- | --- |
| Affx-501395681 | AX-197986815 | ITS2_1 | GATGGCCTCTTGAACGTGCATTGCGCTCTTGGGAT**[A/-]**TGCCTGAGAGCATGTCTGCTTCAGTGCTTCTACTT | [S/BCD] |
| Affx-501395682 | AX-197986817 | nr28S_1 | TAAGCATATAAGTAAGCGGAGGAAAAGGAACTAAA**[C/T]**AGGATTCCCTTAGTAATGGCGAACGAACAGGGATC | [SD/BC] |
| Affx-501395683 | AX-197986819 | nr28S_2 | CAGCAACCGACCAATCAATTGGGAGAAGTTTGAGT**[A/T]**AGAGCATGTGTGTTAGGACCCGAAAGATGGTGAAC | [SD/BC} |
| Affx-501395694 | AX-285063924, AX-285063915, AX-285063919 | elf2_1 | TACCTGATTGAGATCAAGGAGCATGTGAACAGCGC**[G/T/C]**TTCCAGTGGGCCACCAAGGAAGGACCTCTGTGCGA | [S/B/CD] |
| Affx-501395684 | AX-197986821 | cp23S_1 | ATAACGGTCCTAAGGTAGCAAATTTCCTTGTCGTC**[C/T]**TAATAACGACCTGCATGAAACATAGAACGATTCGA | [SCD/B] |
| Affx-501395685 | AX-197986822 | cp23S_2 | AAGTGCAAAGATACATGTTTCGCTTAATGGCCCAA**[T/-]**GAAGTCCTTCCCAGTATTTAAATGCTATCTTAATG | [D/SCB] |
| Affx-501395698 | failed | psbA_1 | CTTTATGGCAACAACATTATAACAGGAGCTGTAAT**[T/C/A]**CCGAGTTCTAATGCTATTGGTGTTCATTTCTATCC | [SD/B/C] |
| Affx-501395686 | AX-197986824 | psbA_2 | TGCTTATATAATGGTGGAACATATCAATTTGTAGT**[C/A]**CTTCACTTCATGCTTGGTGTGGCTTGCTGGATGGG | [SB/CD] |
| Affx-501395702 | AX-285063925, AX-285063929 | psbA_3 | TTTGGTCAAGAAGATGAAACTTATAGCATATCAGC**[T/C/A]**GCTCATGGTTATTTTGGTAGACTCATATTTCAATA | [S/B/CD] |
| Affx-501395706 | AX-285063939, AX-285063930 | COI_1 | CCTAGAGTCAATAATTTTTCTATCTTAATTCTTTT**[A/C/G]**CTTTCATATCTTTTCCTAATCCTTTCTATAATCTC | [S/B/CD] |
| Affx-501365411 | AX-198034302, AX-197937519 | COI_2 | TTCATGCTTTTATTAACATTACCAATCTTATCTGG**[T/A]**ACACTTCTTTTAATATTGGGTGATCTTCATTCTAA | [SBC/D] |
| Affx-501395690 | AX-285063938, AX-285063914, AX-285063940 | cob_1 | TTAAGGAATTCCACTAATAATAAAATAGCATTTTT**[T/G/C]**CCTTTCATTATTAGTAAAGATTTCTATGGAAAGAT | [S/B/CD] |

* S= *Symbiodinium*, B= *Breviolum*, C= *Cladocopium*, D= *Durusdinium*

**Table S11. Number of population probes by species and location.**

| Pairwise Comparison | *A. palmata* population probes | *A. cervicornis* population probes |
| --- | --- | --- |
| VI to CU | 1,026 | 617 |
| VI to BE | 1,727 | 1,531 |
| VI to FL | 751 | 656 |
| CU to FL | 532 | 607 |
| CU to BE | 1,530 | 1,338 |
| FL to BE | 253 | 586 |

**Table S12. Sequence accession ID for genera probe design.**

| Gene | Accessions | References |
| --- | --- | --- |
| cob | JN557965.1, JN557953.1,  JN557943.1, JN557956.1, JN557957.1 | ^5^ |
| COI | JN557913.1, JN557901.1, JN557891.1, JN557904.1, JN557905.1 | ^5^ |
| cp23S | JN558021.1, JN557991.1, JN557969.1, JN558007.1, JN558010.1 | ^5^ |
| elf2 | JN557889.1, JN557879.1, JN557869.1, JN557882.1,  JN557883.1 | ^5^ |
| nr28S | JN558091.1, JN558057.1,  JN558040.1, JN558075.1 | ^5^ |
| ITS2 | AF333507.1, AF333511.1, AF499787.1, AF180124.1, DQ480600.1, AF499793.1, AF499797.1, AF334660.1, | ^6-9^ |
|  | Arif *et al.* ITS2 database |  |
| psbA | JN557866.1, JN557854.1, JN557844.1, JN557857.1, AB086863-AB086880.1 | ^5,10^ |


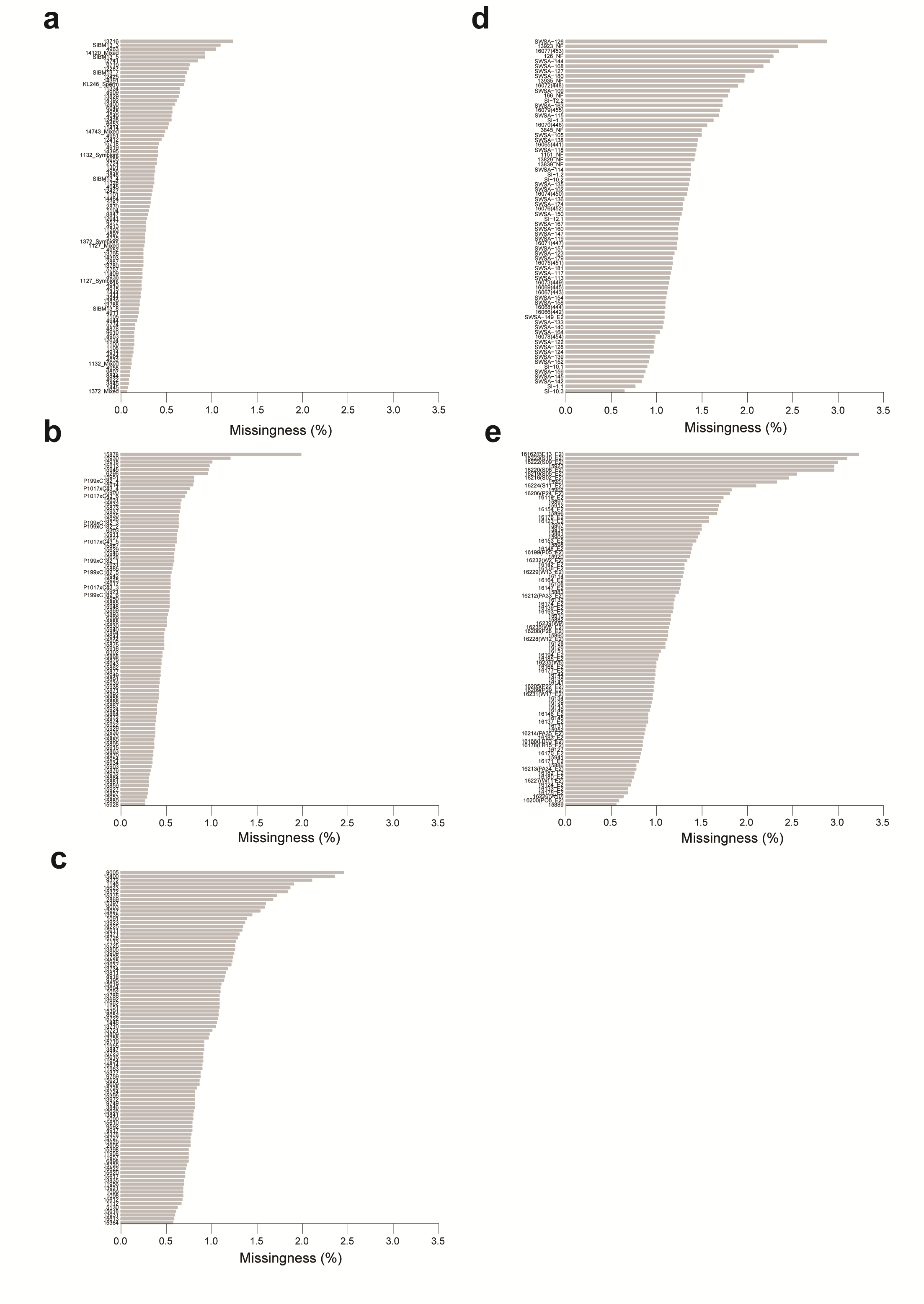


**Figure S1. Percentage of missing genotype calls per sample split by each plate.** Plates P9SR10073 (a), P9SR10074 (b), P9SR10076 (c), 9SR22843 (d) and 9SR22844 (e).


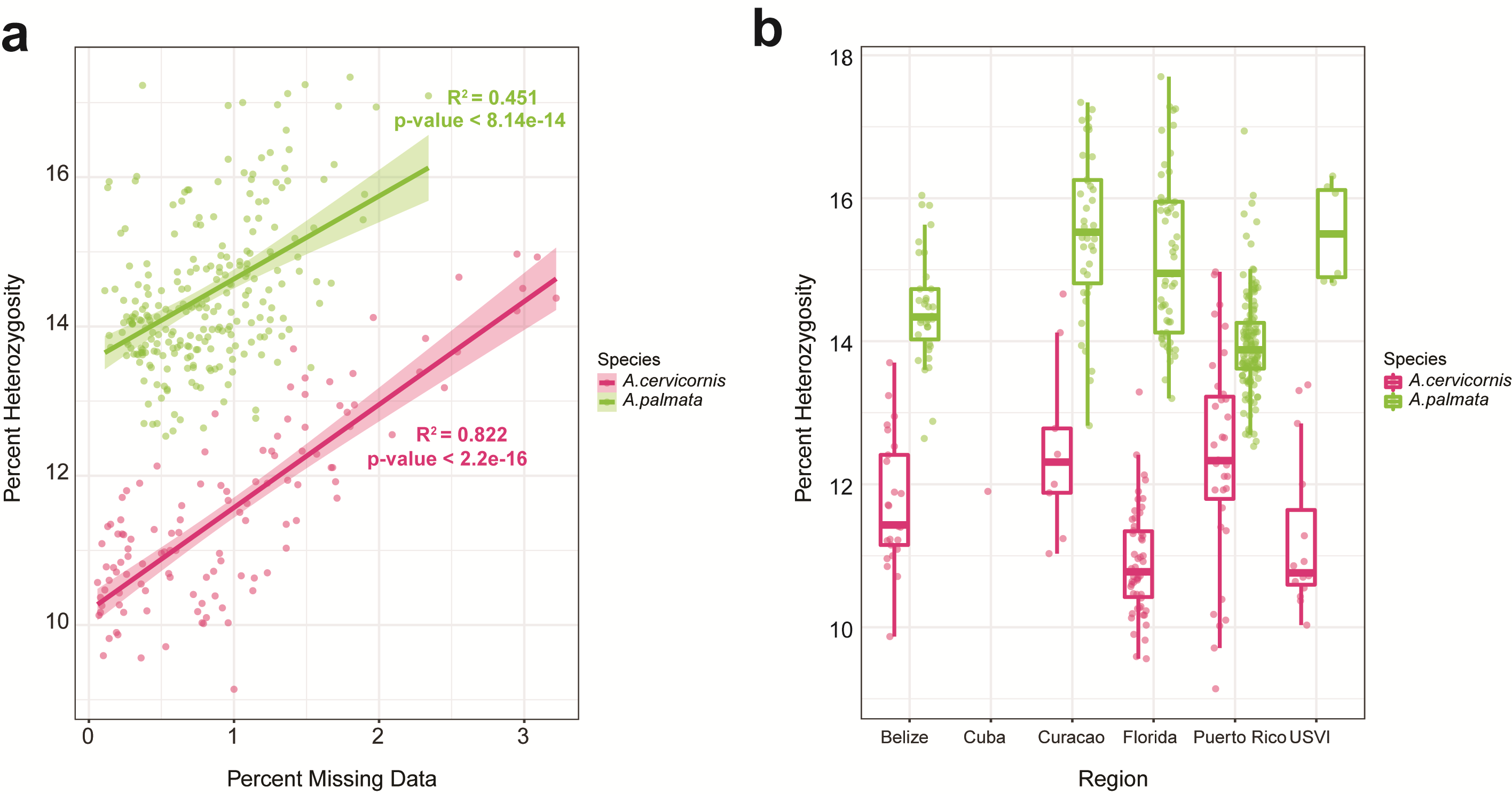


**Figure S2. Percentage of heterozygosity by species and geographic region.** A positive correlation was detected between percentage of missing data and heterozygosity for each species (a). A breakdown by collection location and species reveals higher total percent heterozygosity in *A. palmata* compared to *A. cervicornis* (b).

**
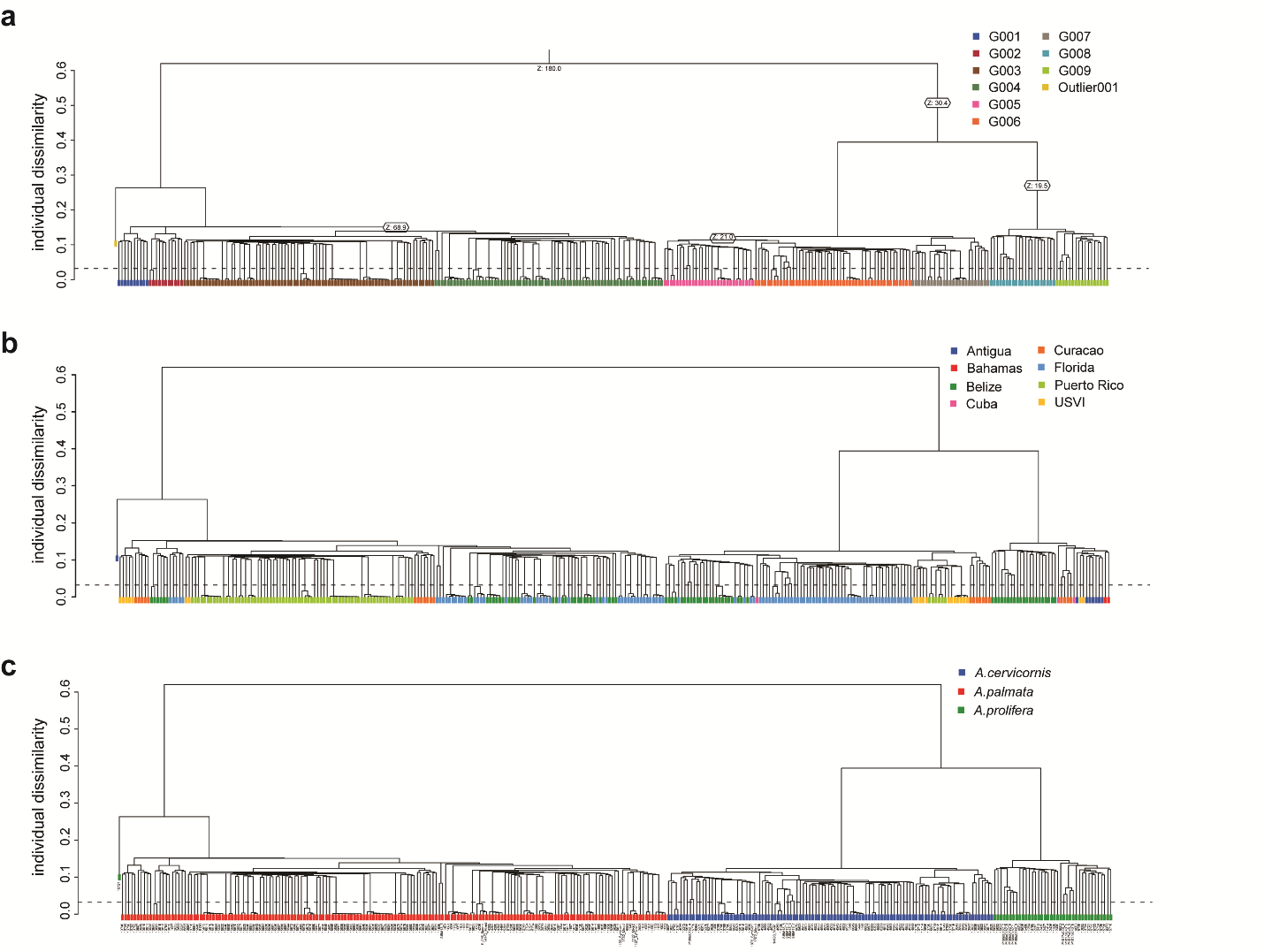
**

**Figure S3. Identity-by-state clustering for three plates based on z-score (a), region (b) or species (c).**

**
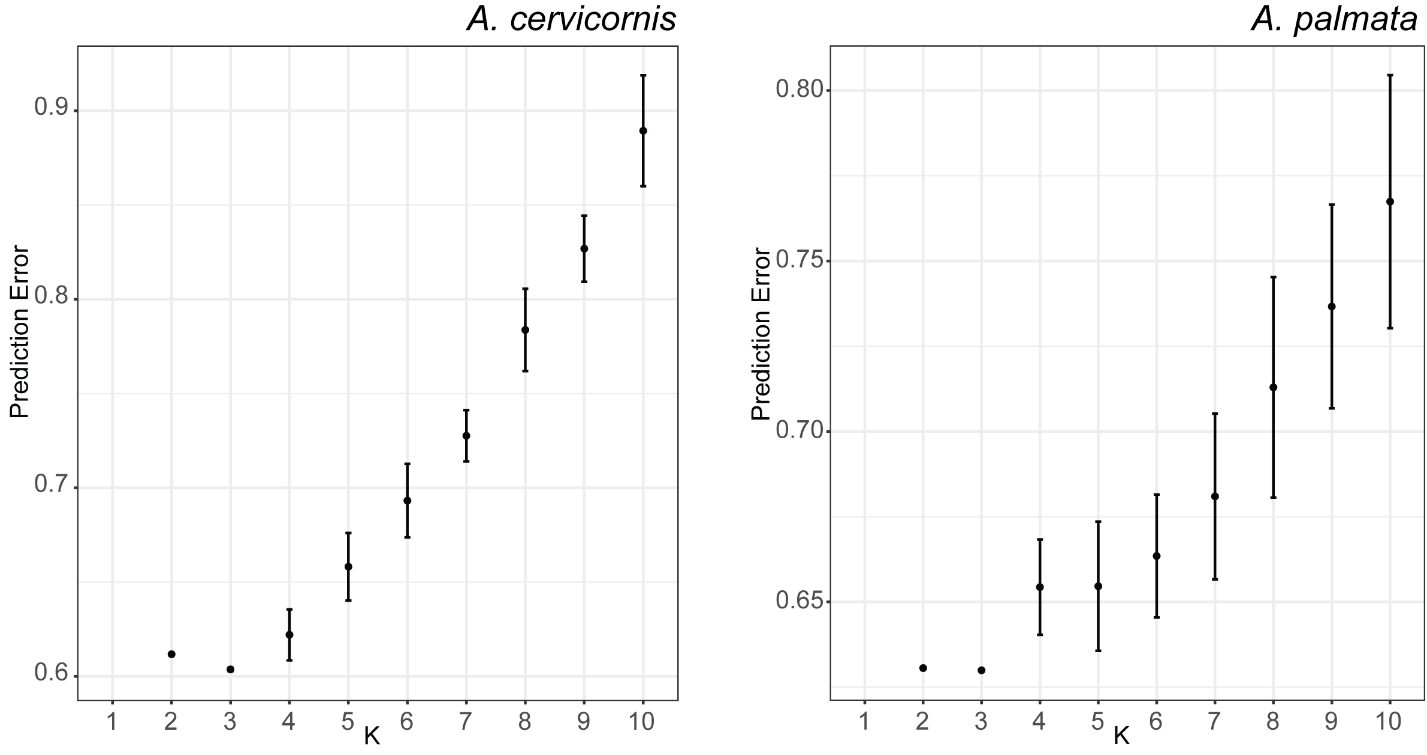
**

**Figure S4. Cross-validation error of tested K populations.** Each value of K was repeated 20 times with a different random seed in ADMIXTURE. The mean value of CV prediction error +/- the standard deviation is shown. K=3 had the lowest CV errors for both species (*A. cervicornis* = 0.604 ± 0.0009 and *A. palmata* = 0.630 ± 0.0002).


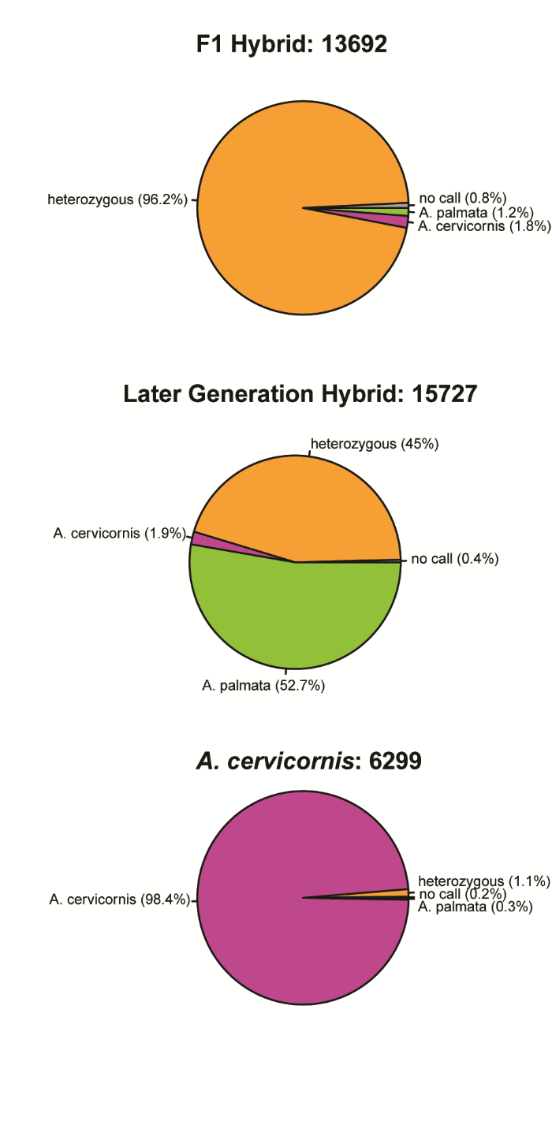


**Figure S5.** **Species-specific SNPs identify hybrids.** Sample 13692 was identified as an F1 and sample 15727 as a later generation hybrid. For comparison, sample 6299 is identified as a pure *A. cervicornis* sample. The 9,072 fixed SNPs were scored as homozygous for each species, *A. palmata* or *A. cervicornis*, or as heterozygous.

**
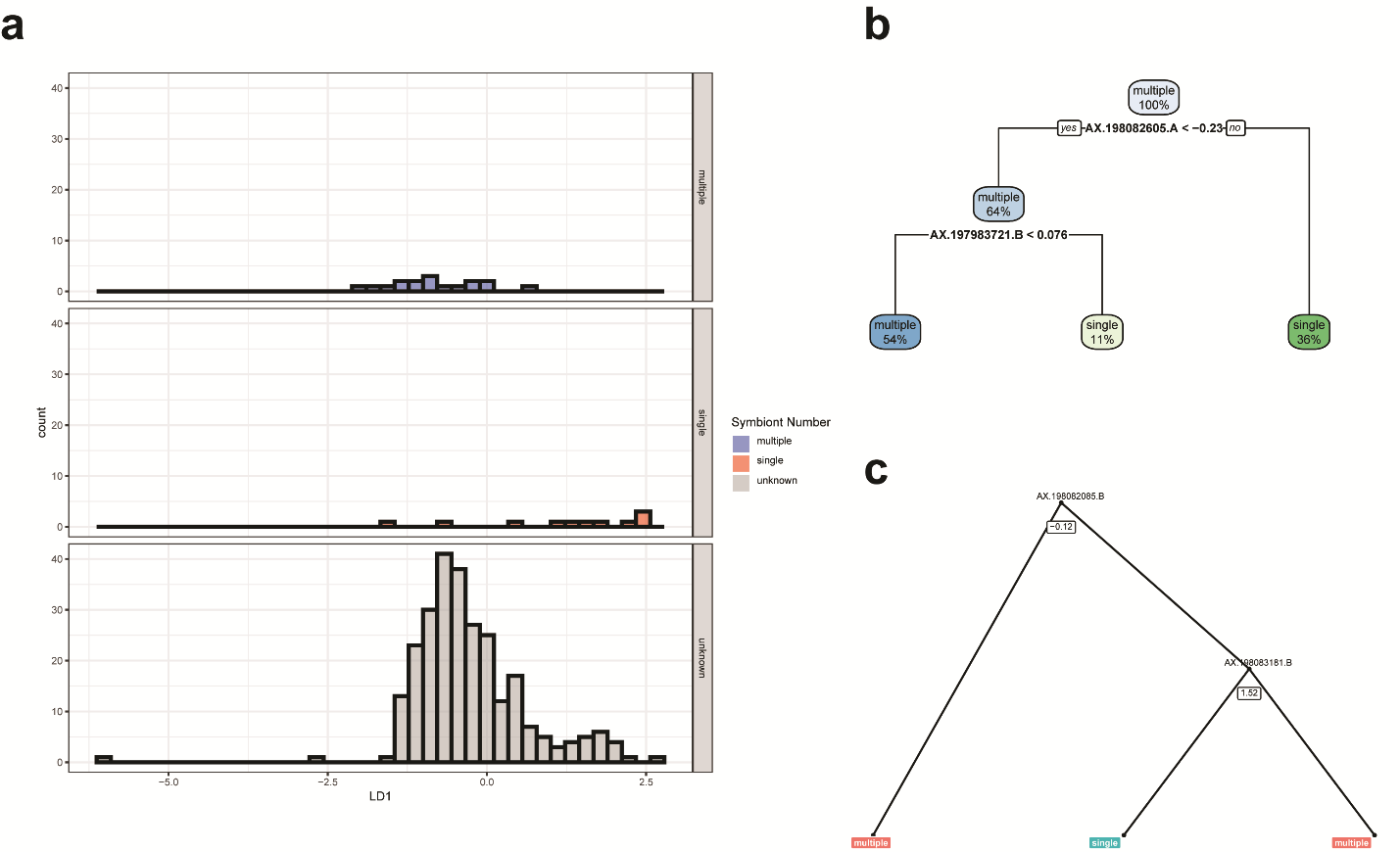
**

**Figure S6. Single or multiple symbiont colonization. Linear discriminant analysis (a) decision tree (b) and random forest example tree (c).**

**
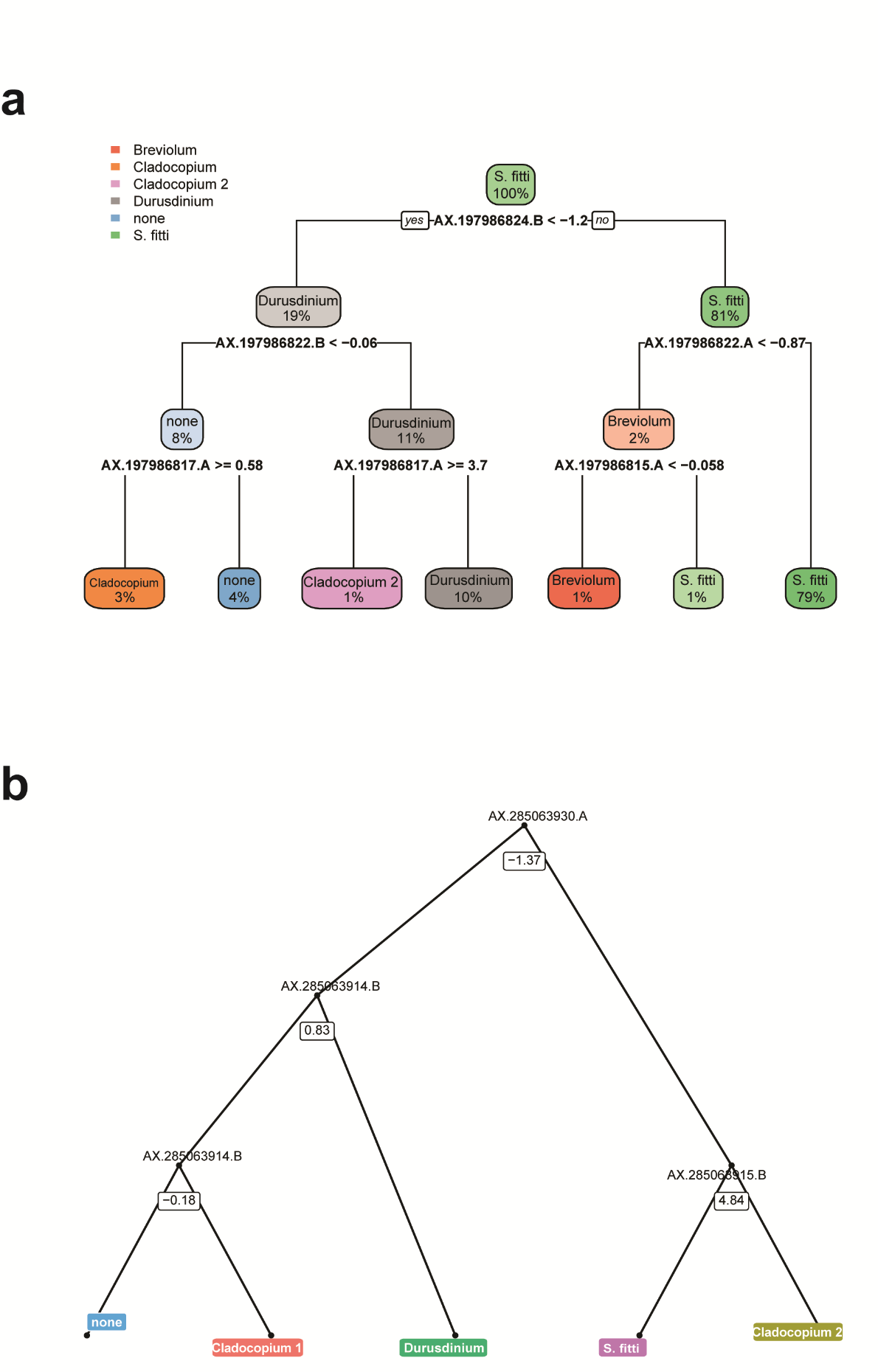
**

**Figure S7. Decision tree (a) and random forest tree (b) with the lowest error rate and maximum nodes for symbiont genera assignment.**

**
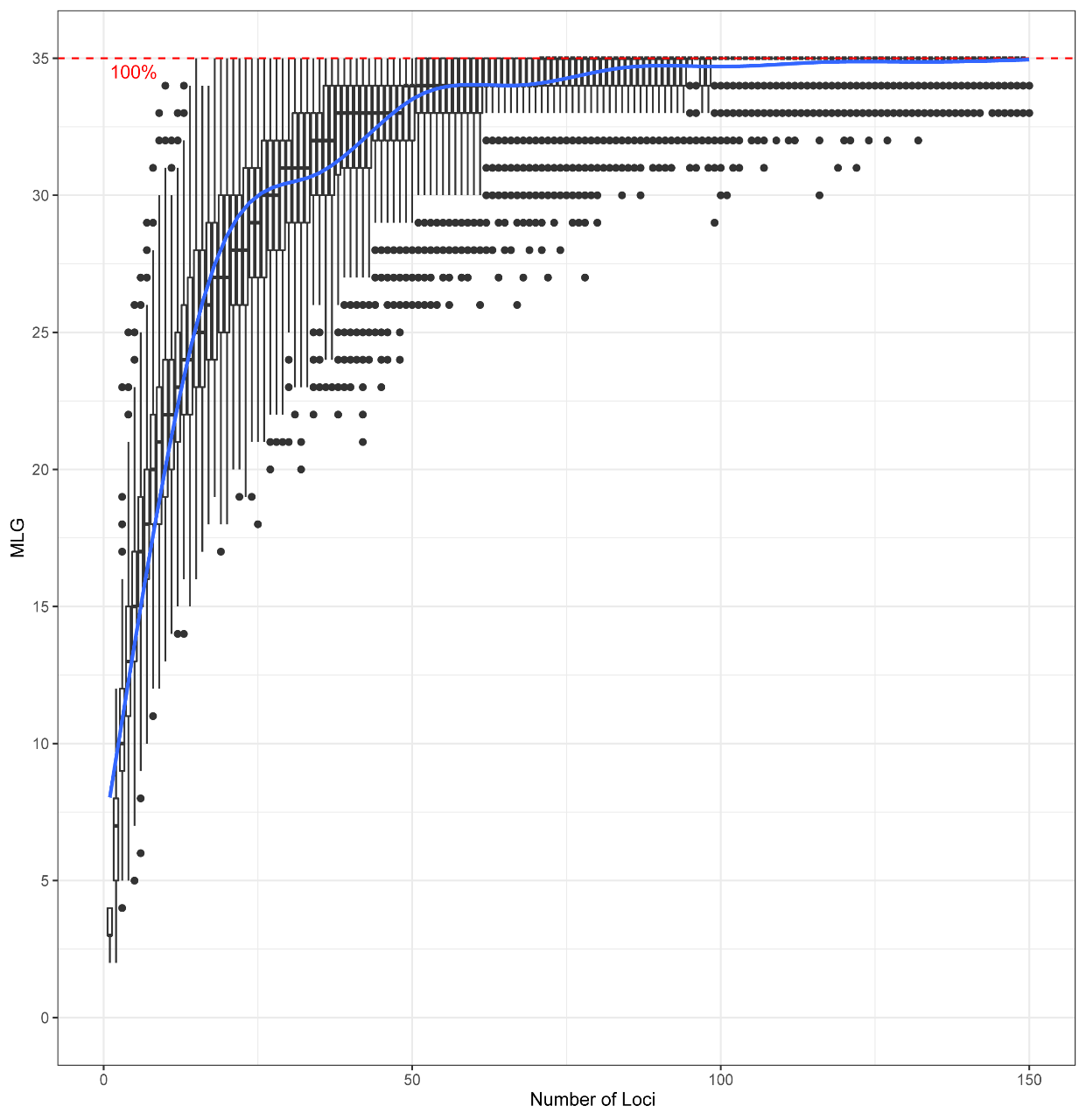
**

**Figure S8. Genotype accumulation curve of the Pacific samples.** The minimum number of loci required to recover 35 unique genet IDs. Boxplots are the results of the number of loci on the x-axis randomly sampled 100 times from all loci.


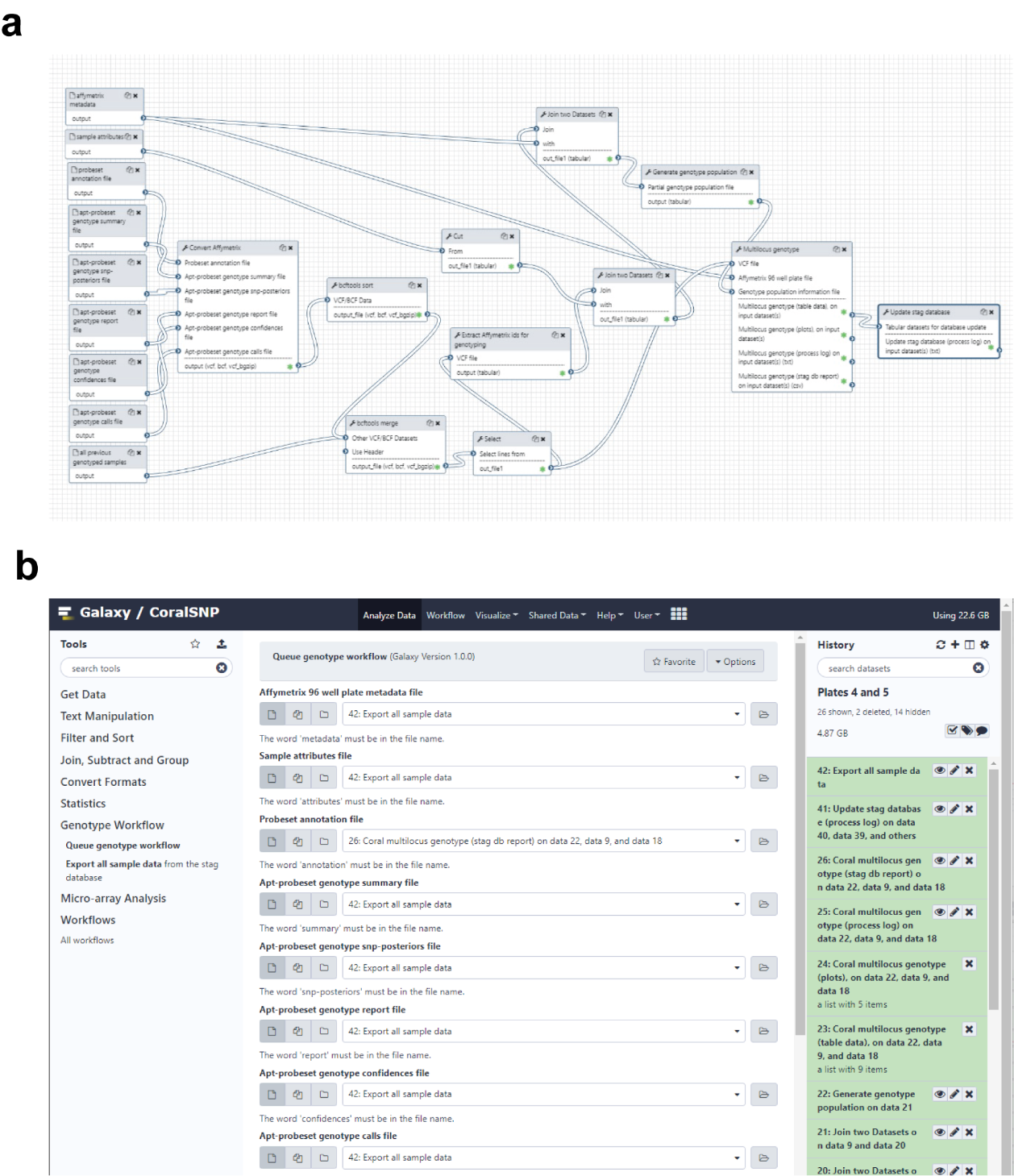


**Figure S9. Galaxy CoralSNP workflow and interface.** This analysis pipeline (a) is initiated by the *Queue Genotype Workflow* tool via the Galaxy REST API. The workflow consists of the following tools, all of which can be installed into a local Galaxy instance from the Main Galaxy Tool Shed (<https://toolshed.g2.bx.psu.edu>). Affy2vcf2 converts Affymetrix genotype calls and intensity files to the VCF format. Bcftools_sort sorts bcf/vcf files. Bcftools_merge merges bcf/vcf files. Affy_ids_for_genotyping extracts information from a VCF files that contains Affymetrix identifiers and produces a file that contains a subset of the identifiers combined with additional data to generate the genotype population information for use as input to the *Coral Multilocus Genotype* tool. Genotype_population_info generates the genotype population information file for use as input to the *Coral Multilocus Genotype* tool. Coral_multilocus_genotype renders the unique combination of alleles for two or more loci for each individual. Update_stag_database updates the stag database tables from a dataset collection where each item in the collection is a tabular file that will be parsed to insert rows into a table defined by the name of the file. The code for these tools is available in GitHub at <https://github.com/gregvonkuster/galaxy_tools/tree/master/tools/corals>. The Galaxy CoralSNP *Queue Genotype Workflow* tool interface (b) consist of the analysis tools in the left tool panel. Selecting a tool displays the tool form in the center panel where the user can select the appropriate inputs for the tool and execute it. The tool outputs are added to the Galaxy analysis history on the right. The *Queue Genotype Workflow* tool accepts eight data files as inputs, the user metadata file and the Affymetrix sample attributes, annotation, summary, snp-posteriors, report, confidences and calls files. The tool includes a reference genome selection for the analysis. Once the tool is executed, the user can simply wait for the CoralSNP analysis pipeline to finish in the right panel.

**
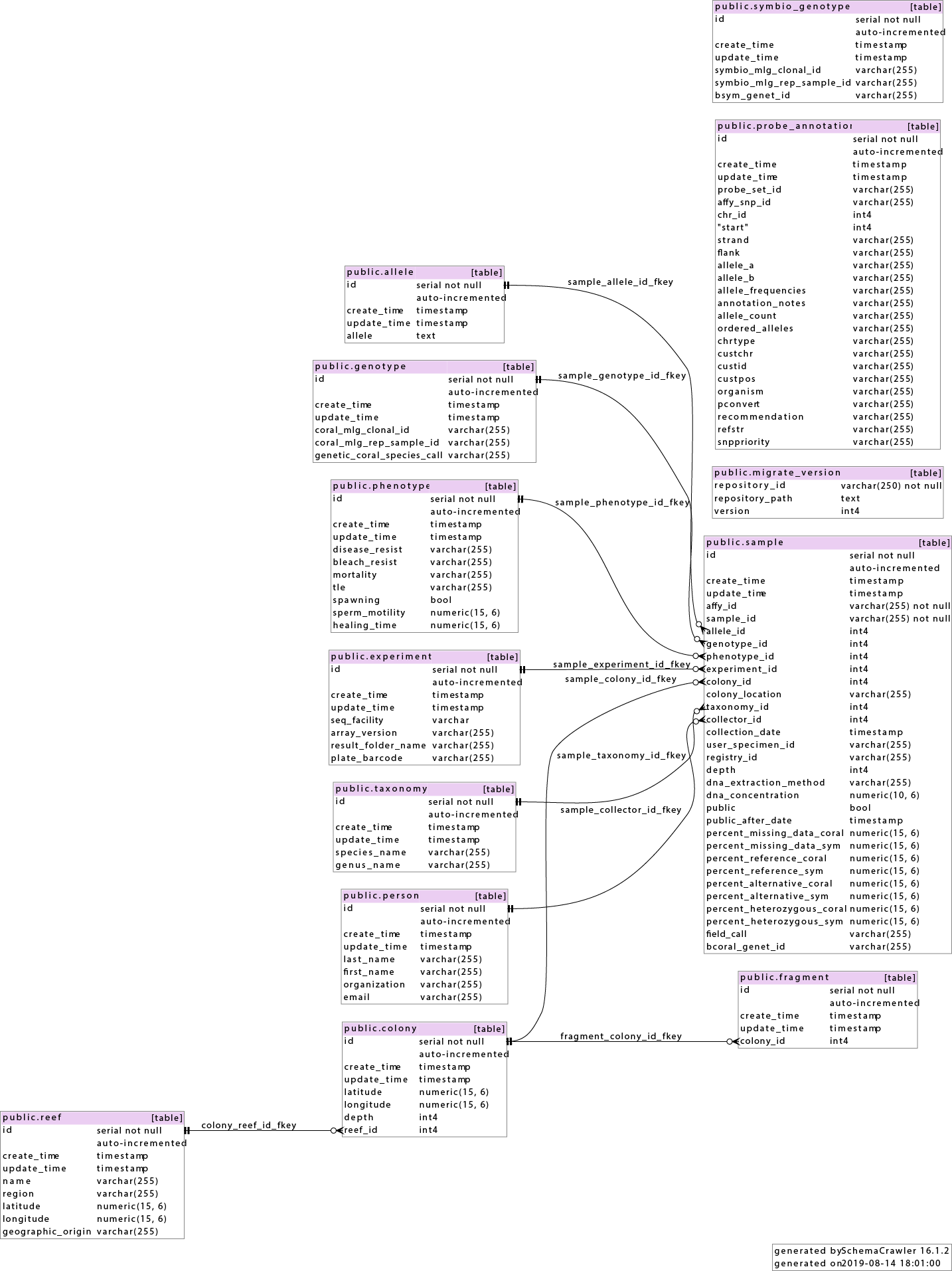
**

**Figure S10. STAG database schema.** This database was initially populated with the genotypes of 42 acroporid genomes that were sequenced in 2017. The database contains the genotype pattern for each unique clonal ID and a list of all samples matching that clonal ID. It also contains metadata provided by the user about each sample such as collection site (GPS), collection date, sample depth, contact information of the collector, and sequencing facility of the raw data.

**References**

1 Fukami, H. *et al.* Geographic differences in species boundaries among members of the *Montastraea annularis* complex based on molecular and morphological markers. *Evolution* **58**, 324-337 (2004).

2 Levitan, D. R., Fogarty, N. D., Jara, J., Lotterhos, K. E. & Knowlton, N. Genetic, spatial, and temporal components of precise spawning synchrony in reef building corals of the *Montastraea annularis* species complex. *Evolution: International Journal of Organic Evolution* **65**, 1254-1270 (2011).

3 Wilson, K. *et al.* Genetic mapping of the black tiger shrimp *Penaeus monodon* with amplified fragment length polymorphism. *Aquaculture* **204**, 297-309 (2002).

4 Bongaerts, P. *et al.* Deep reefs are not universal refuges: reseeding potential varies among coral species. *Science Advances* **3**, e1602373 (2017).

5 Pochon, X., Putnam, H. M., Burki, F. & Gates, R. D. Identifying and characterizing alternative molecular markers for the symbiotic and free-living dinoflagellate genus *Symbiodinium*. *PLoS One* **7**, e29816 (2012).

6 LaJeunesse, T. C. Investigating the biodiversity, ecology, and phylogeny of endosymbiotic dinoflagellates in the genus *Symbiodinium* using the ITS region: in search of a “species” level marker. *J. Phycol.* **37**, 866-880 (2001).

7 Reimer, J. D., Takishita, K. & Maruyama, T. Molecular identification of symbiotic dinoflagellates (*Symbiodinium* spp.) from *Palythoa* spp.(Anthozoa: Hexacorallia) in Japan. *Coral Reefs* **25**, 521-527 (2006).

8 LaJeunesse, T. C. Diversity and community structure of symbiotic dinoflagellates from Caribbean coral reefs. *Mar. Biol.* **141**, 387-400 (2002).

9 Arif, C. *et al.* Assessing *Symbiodinium* diversity in scleractinian corals via next-generation sequencing-based genotyping of the ITS2 rDNA region. *Mol. Ecol.* **23**, 4418-4433, doi:10.1111/mec.12869 (2014).

10 Takishita, K., Ishikura, M., Koike, K. & Maruyama, T. Comparison of phylogenies based on nuclear-encoded SSU rDNA and plastid-encoded psbA in the symbiotic dinoflagellate genus *Symbiodinium*. *Phycologia* **42**, 285-291 (2003).
